# Intra-subject stability of different expressions of spatial QRS-T angle and their relationship to heart rate

**DOI:** 10.3389/fphys.2022.939633

**Published:** 2022-08-30

**Authors:** Irena Andršová, Katerina Hnatkova, Ondřej Toman, Martina Šišáková, Peter Smetana, Katharina M. Huster, Petra Barthel, Tomáš Novotný, Georg Schmidt, Marek Malik

**Affiliations:** ^1^ Department of Internal Medicine and Cardiology, University Hospital Brno, Brno, Czech; ^2^ Department of Internal Medicine and Cardiology, Faculty of Medicine, Masaryk University, Brno, Czech; ^3^ National Heart and Lung Institute, Imperial College, London, England; ^4^ Wilhelminenspital der Stadt Wien, Vienna, Austria; ^5^ Klinikum Rechts der Isar, Technische Universität München, Munich, Germany

**Keywords:** healthy volunteers, long-term ECG, ECG measurements, spatial QRS-T angle, heart rate, heart rate hysteresis, polynomial regression, sex differences

## Abstract

Three-dimensional angle between the QRS complex and T wave vectors is a known powerful cardiovascular risk predictor. Nevertheless, several physiological properties of the angle are unknown or poorly understood. These include, among others, intra-subject profiles and stability of the angle relationship to heart rate, characteristics of angle/heart-rate hysteresis, and the changes of these characteristics with different modes of QRS-T angle calculation. These characteristics were investigated in long-term 12-lead Holter recordings of 523 healthy volunteers (259 females). Three different algorithmic methods for the angle computation were based on maximal vector magnitude of QRS and T wave loops, areas under the QRS complex and T wave curvatures in orthogonal leads, and weighted integration of all QRS and T wave vectors moving around the respective 3-dimensional loops. These methods were applied to orthogonal leads derived either by a uniform conversion matrix or by singular value decomposition (SVD) of the original 12-lead ECG, giving 6 possible ways of expressing the angle. Heart rate hysteresis was assessed using the exponential decay models. All these methods were used to measure the angle in 659,313 representative waveforms of individual 10-s ECG samples and in 7,350,733 individual beats contained in the same 10-s samples. With all measurement methods, the measured angles fitted second-degree polynomial regressions to the underlying heart rate. Independent of the measurement method, the angles were found significantly narrower in females (*p* < 0.00001) with the differences to males between 10^o^ and 20^o^, suggesting that in future risk-assessment studies, different angle dichotomies are needed for both sexes. The integrative method combined with SVD leads showed the highest intra-subject reproducibility (*p* < 0.00001). No reproducible delay between heart rate changes and QRS-T angle changes was found. This was interpreted as a suggestion that the measurement of QRS-T angle might offer direct assessment of cardiac autonomic responsiveness at the ventricular level.

## 1 Introduction

The observations that the myocardial repolarisation sequence cannot follow the same spatial path as the preceding depolarisation sequence was made not long after the invention of electrocardiography. Already in 1934, Wilson et al. (1934) proposed the measurements of areas under the electrocardiogram (ECG) deflections to quantify the disparity between spatial orientations of the QRS complex and the T wave. This disparity was subsequently refined by Geselowitz (Geselowitz, 1983) who proposed mathematical forms of the so-called ventricular gradient. The concept was subsequently further elaborated but with little practical implications. Indeed, in 1989, Macfarlane and Lawrie wrote in their substantial ECG book (Macfarlane and Lawrie, 1989) that “the most exciting thing about the ventricular gradient is its name”.

In 2000, however, the seminal report by Zabel et al., (2000) showed that the angle of the ventricular gradient, that is the 3-dimensional angle between the QRS and T wave orientations, is a potent predictor of mortality risk in survivors of acute myocardial infarction. Subsequently, the risk stratification strength of the QRS-T angle has been confirmed in a large number of studies including investigations of other ischaemic heart disease populations (de Torbal et al., 2004; Malik et al., 2004), acute coronary syndrome (Lown et al., 2012), heart failure (Gotsman et al., 2013; Selvaraj et al., 2014; Sweda et al., 2020), hypertrophic (Cortez et al., 2017a; Cortez et al., 2017b; Jensen et al., 2021) and dilated cardiomyopathy (Li et al., 2016), diabetic patients (Voulgari et al., 2010; Cardoso et al., 2013; May et al., 2017; May et al., 2018), renal patients on haemodialysis (de Bie et al., 2013; Poulikakos et al., 2018); and many other populations and conditions ranging from systemic sclerosis (Gialafos et al., 2012) and Chagas disease (Zampa et al., 2014) to overall hospital (Yamazaki et al., 2005) and general populations (Kardys et al., 2003; Kors et al., 2003; Walsh et al., 2013). It has also recently been shown that QRS-T angle might be meaningfully combined with other ECG-based risk factors (Hnatkova et al., 2022).

The risk-prediction properties of the QRS-T angle are thus now proven beyond any reasonable doubt. The strength of the QRS-T angle-based prediction of cardiovascular risk and/or of cardiovascular death has been shown comparable if not exceeding that of established risk factors including left ventricular ejection fraction, QRS complex width, and heart rate (Hnatkova et al., 2022). Nevertheless, several physiological aspects of the parameter are either unknown or poorly understood. While population-based relationship of the angle to the underlying heart rate has previously been reported (Smetana et al., 2004; Kenttä et al., 2010), little is known about the individual intra-subject profiles of this relationship and of its relationship to sex and age. Simple comparison with the hysteresis loops of the QT interval adaptation to the changes in the underlying heart rate suggested that the QRS-T angle changes follow the heart rate changes more rapidly (Hnatkova et al., 2010) but no details on the possible angle/heart-rate hysteresis are known. Practical comparisons of different possibilities of calculating the angle were reported (Hnatkova et al., 2018) but it is unknown whether these possibilities differ in the intra-subject reproducibility when the relationship to heart rate is considered.

Concentrating on these knowledge gaps, we utilised ECG measurements made in long-term 12-lead Holter recordings obtained in a large population of normal healthy volunteers who were investigated during clinical pharmacology studies. For the comparison of the possibilities of the angle calculations, we used three different methods, namely the measurements based on the so-called maximum and area calculations (Cortez and Schlegel, 2010; Oehler et al., 2014) as well as a measurement variant derived from the initial reports of the clinical usefulness of the angle measurement (Zabel et al., 2000). The initial report of the risk-prediction value expressed the angle as an integral of cosines of angles between the QRS complex directions and the maximum T wave direction. Since this was not mathematically consistent, we introduce a method that uses the integral method applied to both the QRS complex and the T wave directions.

## 2 Materials and methods

### 2.1 Investigated population and electrocardiographic recordings

The ECG recordings used in the study have been reported previously in studies that investigated very different electrophysiology factors (Hnatkova et al., 2021; Andršová et al., 2022; Toman et al., 2022). For the purposes of the present investigation, we applied very different data processing methods.

In brief, clinical pharmacology studies were conducted at 3 different clinical research sites. These studies enrolled 523 healthy volunteers including 259 females. Before study enrolment, all the volunteers had a normal standard clinical ECG and normal clinical investigation. All the source studies used the same standard inclusion and exclusion criteria mandated for Phase I pharmacology investigations (ICH Guideline, 2001) including negative recreational substances tests and negative pregnancy tests for females. All the source studies were ethically approved by the institutional ethics bodies (Parexel in Baltimore; California Clinical Trials in Glendale; and Spaulding in Milwaukee). All subjects gave informed written consent to study participation including scientific investigation of collected data.

Demographic data including age, sex, racial classification, body height, and body weight were collected. Lean body mass (LBM) was calculated as LBM = 0.29569*W+41.813*H-43.2933 for females and LBM = 0.3281*W+33.929*H-29.5336 for males, where W is body weight in kilograms and H is body height in metres (Hume, 1966); it was expressed in kilograms. Body mass index (BMI) was calculated as BMI = W/H^2^.

In each study participant, repeated three to four long-term 12-lead Holter ECG recordings with Mason-Likar electrode positions were obtained during full day-time periods (i.e., each recording was approximately 14–16 h long) while the subjects were not allowed to smoke and/or consume alcohol or caffeinated drinks and/or to sleep. No medication was administered during these recordings and, where appropriate, sufficient wash-out periods after previous treatment periods were used. The study protocols were mutually consistent in respect of the conduct during the drug-free baseline days. Since only drug-free data were used in the investigation reported here, further details of source studies are immaterial.

As previously reported (Malik et al., 2008a; Malik et al., 2012), multiple non-adjacent 10-s ECG segments were extracted from the long-term ECGs. All these 10-s segments contained only sinus rhythm free of ectopic beats and were extracted (a) from pre-specified time-points of the source pharmacologic studies, (b) from recording scans aimed at finding representative spectrum of different underlying heart rates of selected ECG segments distinguishing those that were and were not preceded by heart rate changes exceeding ±2 beat per minute. That is, the complete day-time recordings were searched to identify ECG segments that were preceded by stable or variable heart rates and the extractions were made from these segments while keeping the distinction between stable and variable preceding rates. The ECG segments were pre-selected for further processing only if satisfactory algorithmic measurement of QT interval was possible (Malik et al., 2008a; Malik et al., 2012). This was judged by similarities in the measured QT intervals of partially overlapping and adjacent ECG segments. Nevertheless, since all ECG interval measurements were subsequently visually verified and, where appropriate, manually corrected (see the next section) the judgement details of algorithmic measurements are of no importance.

### 2.2 Electrocardiographic measurements

In each of these ECG segments, QRS complex (i.e., the Q wave onset and J point) and the T wave offset were identified following published procedures (Malik et al., 2008a; Malik et al., 2012) that included repeated visual controls, manual corrections of all the measurements, and intra- and inter-subject repeatability and stability of the measurements. Consistency of the interpretation of corresponding ECG morphologies was also assured (Hnatkova et al., 2009). The visually verified QT interval measurements were made in the representative median waveforms of the 10-s segments (sampled at 1,000 Hz) with the superimposition of all 12 leads on the same isoelectric axis (Malik, 2004; Xue, 2009).

Using a previously proposed technique (Berger et al., 1997; Baumert et al., 2008), QT interval was projected to individual beats within the 10-s ECG by finding the maximum correlation between the median waveform and the signal of individual QRS-T complexes. The maximum correlations were identified separately for the surroundings of the QRS onset and of the T wave offset. Since it has previously been observed that this process might lead to slightly different results when applied to different ECG leads (Malik, 2008), the cross-correlation technique was applied to the vector magnitude of algebraically reconstructed orthogonal leads (Guldenring et al., 2012).

Subsequently, Pearson correlation coefficients were calculated between the analysed beat and representative median waveform in (a) a window surrounding the QRS onset by ±40 ms and (b) a window surrounding the T wave offset by ±50 ms. The QRS-T pattern of the given beat was accepted for further analysis only if both these correlation coefficients exceeded 0.9. This assured that beats substantially distorted by pollution noise were excluded. No other restrictions were applied. This made the data selection/exclusion less stringent compared to previous investigations of beat-to-beat QT interval analyses (Toman et al., 2022).

The source clinical studies included episodes of per-protocol changes of postural positions. This allowed to capture measurable ECG segments at broad heart rate ranges (Malik et al., 2012).

### 2.3 RR interval histories

For each analysed ECG segment, a 5-min history of preceding RR intervals was obtained. This was combined with the positions of the individual beats within the segment, allowing to obtain a beat-specific history of RR intervals in a 5-min history of each analysed beat. That is, the RR interval of the histories also included the positions of all QRS complexes regardless of whether these were or were not accepted for further analysis.

### 2.4 Expressions of spatial QRS-T angle

#### 2.4.1 Orthogonal leads

The assessment of spatial QRS-T angle requires to derive 3 orthogonal leads from the 12-lead ECG signal. This was performed in two different ways.

Firstly, previously published conversion matrix suitable for Mason-Likar electrode positions (Guldenring et al., 2012) was used to derive orthogonal leads XYZ for each of the median representative waveforms and for each QRS-T beat accepted for further analysis.

Subsequently, the same 12-lead ECG signals (i.e., the representative waveforms and the beats accepted for analysis) were processed by singular value decomposition (Acar et al., 1999; Acar and Köymen, 1999; Yana et al., 2006; Hnatkova et al., 2021) between the QRS onset and T wave offset. This derived three dominant and mutually orthogonal leads S_1_, S_2_, and S_3_ (as if the system of orthogonal axes was optimally rotated for the given 12-lead signal).

#### 2.4.2 QRS-T angle measurement

Three principal algorithmic methods of deriving the 3-dimensional QRS-T angle were considered. The following notations is used in their descriptions:

A triplet of values [φ,ϕ,ψ] is considered to represent a vector between the zero point [0,0,0] and the point determined by the triplet. Such a vector will be denoted as B[φ,ϕ,ψ] . Vector magnitude of vector W=B[φ,ϕ,ψ] is given by formula $\sqrt{\varphi^{2}+\phi^{2}+\psi^{2}}$ and will be denoted as ||W|| . Symbol ℧(G,ℍ) will represent the 3-dimensional angle between vectors G and ℍ . Finally, orthogonal XYZ system of ECG leads will be considered defining functions X(t) , Y(t) , and Z(t) , which assign the voltage values of each of the orthogonal leads to each instance t of the ECG recording. A vector B[X(t),Y(t), Z(t)] will be denoted as ℰ(t) .

##### 2.4.2.1 Area method

The area-based QRS-T angle method uses the integrals of the orthogonal ECG leads (van Oosterom, 2014). That is, using the described notation, the orientation of the QRS complex is defined by a vector.

$$V_{QRS}=B\left[\overset{J}{\underset{Q}{\int }}X\left(t\right)\;dt,\overset{J}{\underset{Q}{\int }}Y\left(t\right)\;dt,\;\overset{J}{\underset{Q}{\int }}Z\left(t\right)\;dt\;\right]$$

and similarly, the orientation of the T wave is defined by a vector

$$V_{T}=B\left[\overset{T_{e}}{\underset{J}{\int }}X\left(t\right)\;dt,\overset{T_{e}}{\underset{J}{\int }}Y\left(t\right)\;dt,\;\overset{T_{e}}{\underset{J}{\int }}Z\left(t\right)\;dt\;\right]$$

where Q , J , and $T_{e}$ specify QRS onset, QRS offset, and T wave offset, respectively. The 3-dimensional QRS-T angle is subsequently computed as the spatial angle $℧\left(V_{QRS},V_{T}\right)\;$ between these two vectors.

##### 2.4.2.2 Maximum method

The maximum vector-based QRS-T angle calculation is based on the notion that QRS and T wave orientations are defined by the maxima of the vector magnitudes within the corresponding orthogonal loops (Cortez et al., 2017a). That is, within the orthogonal XYZ system of ECG leads, time points $t_{QRS}$ and $t_{T}$ are selected such that $\|ℰ\left(t_{QRS})\right\|=\underset{Q\leq t\leq J}{\max}\|ℰ\left(t)\right\|$ and similarly $\|ℰ\left(t_{T})\right\|=\underset{J\leq t\leq T_{e}}{\max}\|ℰ\left(t)\right\|$ , where Q , J , and $T_{e}$ again specify QRS onset, QRS offset, and T wave offset, respectively.

The QRS-T angle is then computed as the spatial angle $℧\left(ℰ\left(t_{QRS}\right),ℰ\left(t_{T}\right)\right)\;$ between the maximum magnitude vectors $ℰ\left(t_{QRS}\right)$ and $ℰ\left(t_{T}\right)$ .

##### 2.4.2.3 Integral method

The integral-based QRS-T angle calculation is based on a weighted average of all angles between all points within the QRS loop and all points within the T wave loop, weighted by the product of the magnitudes of the vectors represented by these points. That is, within the orthogonal XYZ system of ECG leads, the integral based QRS-T angle is given by the formula

$$\left(\overset{J}{\underset{t=Q}{\int }}\overset{T_{e}}{\underset{u=J}{\int }}℧\left(ℰ\left(t\right),ℰ\left(u\right)\right)*\left\|ℰ\left(t\right)\right\|*\left\|ℰ\left(u\right)\right\|\;du\;dt\right)/\left(\overset{J}{\underset{t=Q}{\int }}\overset{T_{e}}{\underset{u=J}{\int }}\left\|ℰ\left(t\right)\right\|*\left\|ℰ\left(u\right)\right\|\;du\;dt\right)$$

#### 2.4.3 QRS-T angle expressions

The two possibilities of obtaining orthogonal lead system and the three possibilities of computing the QRS-T angle led to six combinations that were investigated in this study. We shall term these combinations Area_XYZ_, Maximum_XYZ_, Integral_XYZ_, Area_SVD_, Maximum_SVD_, and Integral_SVD_ – all with obvious meanings. All the values of the angles were expressed in degrees with values between 0^o^ and 180^o^.

### 2.5 Data analyses

For each 10-s ECG segment analysed in the study, the six different computations of the QRS-T angle were applied to the median representative waveform and to each of the individual beats accepted for the analysis. For each of the six computations, the values obtained for the individual beats were also averaged.

#### 2.5.1 Comparisons of QRS-T angle expressions

Three different types of comparisons of different QRS-T angle expressions were made. Firstly, for each algorithmic method, the values obtained based on a combination with the XYZ conversion matrix were compared with the values obtained for the same ECG signal based on the combination of the SVD-based orthogonal leads. The comparison was made for both individual beats and representative median waveforms of ECG segments. Secondly, for each of the six QRS-T angle expressions, comparisons were made between the values derived from representative median waveforms of ECG segments and the averages of the values obtained from individual beats within the same ECG segments. Finally, for both the representative median waveforms and individual beats, comparisons were made between Maximum_XYZ_ and Area_XYZ_ results, Maximum_XYZ_ and Integral_XYZ_ results, and Area_XYZ_ and Integral_XYZ_ results. The same comparisons were also made for the three different measurements combined with the SVD-based orthogonal leads.

For each of the comparisons, Bland-Altman-type (Bland and Altman, 1986) of scatter diagram was constructed relating the differences between compared values to their mean. These diagrams were based on data pooled from all ECG segments in all subjects and were judged visually. Cumulative distributions of the pooled differences between compared measurements were also constructed for visual judgement.

To allow statistical evaluation of these comparisons, in each of the analyses and in each of the study subjects, intra-subject mean of absolute differences of the compared QRS-T angle values and intra-subject standard deviation of these differences were also obtained.

#### 2.5.2 Relationship to heart rate

##### 2.5.2.1 Regression model between QRS-T angle and heart rate

For an initial assessment, intra-subject relationships between measured QRS-T angles were investigated based on graphical representations. As an initial approximation of the dependency, second degree polynomial regression model between QRS-T angles and underlying heart rate (or reciprocals of the RR intervals) was used in subsequent investigations.

##### 2.5.2.2 Average estimates of rate hysteresis

To replicate the procedures that were previously used in the estimates of the QT/RR hysteresis (Malik et al., 2016), QRS-T angle measurements in individual beats were related to preceding heart rate derived from different durations of the RR interval history of each beat in which the angle measurement was performed. The duration of the history that leads to the closest fit in the regression analysis approximates the hysteresis constant, i.e., the delay with which the QRS-T angle reacts to heart rate changes.

In more detail, for each individual beat □ at which the QRS-T angle ${\text{Θ}}_{□}$ was measured, underlying heart rate ${ℛ}_{□}\left(d\right)$ was obtained from the average of d RR intervals preceding the beat □ . Subsequently, in each study subject, polynomial regression analysis was evaluated in form

$${\text{Θ}}_{□}=\beta_{0}+\beta_{1}{ℛ}_{□}\left(d\right)+\beta_{2}{ℛ}_{□}^{2}\left(d\right)+\;\varepsilon_{□}$$

Where $\beta_{0}$ , $\beta_{1}$ , and $\beta_{2}$ are subject-specific regression coefficients and $\varepsilon_{b}$ are zero centred regression errors. The coefficient d that led to the lowest regression residual, that is to the minimal value of $\;\sqrt{\sum_{□}\varepsilon_{□}^{2}}$ approximated the heart rate hysteresis of the QRS-T angle Θ.

For this purpose, a geometric progression sequence of 50 values between 1 and 300 was used to vary the coefficient d . Subsequently, standard golden search algorithm was used to determine the “optimal” value of d for each study subject.

The analysis was subsequently repeated deriving the heart rate ${ℛ}_{□}\left(d\right)$ not from d preceding RR intervals but from the RR intervals within the preceding d seconds. Both types of analysis (i.e., heart rate derived from preceding number of RR intervals of the preceding time window) were performed for all QRS-T angle measurements in a study subject as well as in only those measurements that were preceded by variable heart rate.

All these steps were performed for each study subject and for all six QRS-T angle expressions.

##### 2.5.2.3 Exponential decay model of rate hysteresis

The expressions of underlying heart rate based on a simple average of preceding RR intervals does not reflect gradual decrease of the influence of heart rate changes in more remote history. Therefore, exponential decay models were also used, in the same form as previously developed for the purposes of estimating QT/RR hysteresis (Malik et al., 2008b; Malik et al., 2016; Andršová et al., 2022).

That means that the same form of polynomial regression analysis as described with the experiments of average estimates of rate hysteresis (see previous section) were used replacing the ${ℛ}_{□}\left(d\right)$ calculations of underlying heart rate with expressions ${ℋ}_{□}\left(\lambda \right)$ derived from exponential decay models where the parameter λ specifies the speed of the adaptation (see further).

In more detail, if beat □ is preceded by a sequence ${\left\{{\text{RR}}_{i}\right\}}_{i=0}^{N}$ of N consecutive RR intervals ( ${\text{RR}}_{0}$ being the closest to the beat □ ) the underlying heart rate ${ℋ}_{□}\left(\lambda \right)$ is derived from a weighted average of the RR intervals as ${ℋ}_{□}\left(\lambda \right)=60/\overset{N}{\underset{i=0}{\sum }}\omega_{i}{\text{RR}}_{i}$ , where $\overset{N}{\underset{i=0}{\sum }}\omega_{i}=1$ and individual RR intervals are measured in second.

Two different exponential decay models were considered which differed in the definition of the weights ${\left\{\omega_{i}\right\}}_{i=0}^{N}$ . The model assuming the dependency on the number of preceding RR intervals used weights such that

$$\overset{k}{\underset{i=0}{\sum }}\omega_{i}=\frac{1-e^{\lambda \left(\frac{k+1}{N+1}\right)}}{1-e^{\lambda }}$$

for each k,0≤k≤N ,

While the model assuming the dependency on the absolute time preceding the beat □ measurement used the weights such that

$$\overset{k}{\underset{i=0}{\sum }}\omega_{i}=\frac{1-e^{\lambda \left(\frac{T\left(k\right)}{T\left(N\right)}\right)}}{1-e^{\lambda }}$$

where

$$T\left(k\right)=\;\overset{k}{\underset{i=0}{\sum }}{\text{RR}}_{i}$$

for each k,0≤k≤N .

While the parameter λ specifies the speed of the adaptation to heart rate changes, it does not have obvious physiologic interpretation. Therefore, the models were characterised by the so-called hysteresis constant, i.e., by the number of RR intervals or by the time-elapsed at which the adaptation to changed heart rate reaches 95%, i.e., by either a number y of RR intervals or by time delay T(y) such that $\overset{y}{\underset{i=0}{\sum }}\omega_{i}$ = 0.95.

As with the experiments of average estimates of rate hysteresis, the search of optimal subject-specific hysteresis time constant started with a geometric progression sequence of 50 values specifying the hysteresis constant between 1 and 277 (the upper limit determined by the duration of 300 s of the RR interval histories). Subsequently, standard golden search algorithm was used to determine the “optimal” hysteresis constant for each study subject.

#### 2.5.3 Intra-subject characteristics and reproducibility

Physiologic characteristics of QRS-T angle in individual study subjects were investigated in the same way for all six QRS-T angle expressions. For each subject and for each angle expression, setting of the optimal hysteresis constant (and of the optimal interval for heart rate measurement) was used for the assessment of physiologic characteristics. The QRS-T angle measurements at individual beats were therefore used.

##### 2.5.3.1 Curvatures of the relationship to the underlying heart rate

For each subject and for each QRS-T angle expression ${\text{Θ}}^{\left(a\right)}$ , higher degree polynomial regressions were investigated. That is, for different polynomial degree ℘ , regression forms

$${\text{Θ}}_{□}^{\left(a\right)}=\overset{℘}{\underset{i=0}{\sum }}\beta_{i}^{\left(a\right)}{\left[{ℋ}_{□}\left(\lambda \right)\right]}^{i}+\varepsilon_{□}^{\left(a,℘\right)}\;$$

were used, leading to residual error estimates $F^{\left(a,℘\right)}=\sqrt{\sum_{□}{\left(\varepsilon_{□}^{\left(a,℘\right)}\right)}^{2}}$ . Clearly, with increasing degree ℘ , the residual errors decrease, i.e., $F^{\left(a,℘\right)}\geq F^{\left(a,℘+1\right)}$ . The search for the optimum polynomial degree was driven by the criterion $F^{\left(a,℘+1\right)}/F^{\left(a,℘\right)}$ > 0.95 for all six QRS-T angle expression in at least 95% of study population. Polynomial degree ${℘}_{o}$ was defined in this way.

##### 2.5.3.2 Sex and race differences

For each subject and for each QRS-T angle expression ${\text{Θ}}^{\left(a\right)}$ , the ${℘}_{o}$ -degree polynomial regressions provided projections of the ${\text{Θ}}^{\left(a\right)}$ values at heart rate of 60 and 120 beats per minute (bpm). These were subsequently statistically compared between sexes and different subject races.

In addition, for each angle expression (a) , subject-specific polynomial curvatures

$$\overset{{℘}_{o}}{\underset{i=0}{\sum }}\beta_{i}^{\left(a\right)}h^{i}$$

Were computed for h ranging between 50 and 120 bpm and at each value of h (with a step of 1 bpm) median, inter-quartile range, and a 10% to 90% range were calculated for females and males separately.

##### 2.5.3.3 Relationship to Body mass index and Lean body mass

The projections of the ${\text{Θ}}^{\left(a\right)}$ values at heart rate of 60 and 120 bmp were related to body mass index and to lean body mass. The relationship was studied separately in female and male sub-populations.

##### 2.5.3.4 Intra-subject reproducibility

The dependency of QRS-T angle measurements on the underlying heart rate makes it non-sensical to investigate intra-subject reproducibility of the measurements without considering the relationship to heart rate. Therefore, to investigate the reproducibility of the different QRS-T angle expressions, residual errors ${ℭ}^{\left(a,{℘}_{o}\right)}$ of the optimal-degree polynomial regression to the underlying heart rate were used.

##### 2.5.3.5 Slope and curvature of heart rate relationship

In the higher degree polynomial regressions, coefficients $\beta_{i}^{\left(a\right)}$ influence each other. Therefore, to compare the slope of the heart rate relationship between different QRS-T angle expressions, simple linear model was used in the form ${\text{Θ}}_{□}^{\left(a\right)}=\beta_{0}^{\left(a\right)}+\beta_{1}^{\left(a\right)}{ℋ}_{□}\left(\lambda \right)+\;\varepsilon_{□}$ and parameters $\beta_{1}^{\left(a\right)}$ were used to estimate the slope of the heart rate relationship.

The intra-subject degree of the polynomial curvature of the heart rate relationship was estimated by the differences $F^{\left(a,{℘}_{O}\right)}-F^{\left(a,{℘}_{O}-1\right)}$ . That is, after the intra-subject optimum degree ${℘}_{o}$ of the polynomial curvature was established defining the relationship to the hysteresis corrected heart rate, the regression residual of this polynomial curvature was compared to the regression residual of a polynomial curvature in which the polynomial degree was lowered by one.

### 2.6 Statistics and data presentation

Computation of the matrix-based conversion of 12-lead ECG signals to the orthogonal XYZ leads, the SVD of the signals, and the computation of the QRS-T angles utilised purpose developed software written in C++. Microsoft visual studio (version 16.11.8) implementation of Microsoft Visual C++ 2019 compiler (version 00435-60000-00000-AA114) was used.

Descriptive data are presented as means ± SD. Comparison between study groups, e.g., between female and male sub-populations, were tested by two-sample two-tail *t*-test assuming different variances of the compared samples. Intra-subject comparisons, e.g., the comparisons between ${ℭ}^{\left(a,{℘}_{O}\right)}$ and ${ℭ}^{\left(b,{℘}_{O}\right)}$ values, were tested by the paired two-tail *t*-test.

Pearson correlation coefficients and linear regressions were used to study the relationship between BMI and LBM and the QRS-T angle values. The linear regressions were computed together with their 95% confidence bands.

Statistical tests used IBM SPSS package, version 27. *p* values below 0.05 were considered statistically significant. Because of interdependence between the different indices, no correction for multiplicity of statistical testing was made.

## 3 Results

### 3.1 Population and electrocardiographic data

As stated, the source clinical pharmacology studies enrolled 523 healthy volunteers including 259 females. There were no statistical age differences between females (33.4 ± 9.1 years, range 18.1–55.4 years) and males (33.7 ± 7.8 years, range 18.2–54.5 years). Similarly, the body mass index was not different between females (25.2 ± 2.9 kg/m^2^, range 20.0–30.0 kg/m^2^) and males (25.6 ± 2.7 kg/m^2^, range 20.2–30.1 kg/m^2^). As expected, lean body mass was lower in females (45.3 ± 5.0 kg) than in males (57.1 ± 5.3 kg, *p* < 0.0001). Among females, 38.6% and 56.7% of the subjects classified themselves as of African origin and of White Caucasian origin, respectively. Among males, these proportions were 51.3% and 42.8%, respectively. The remainder of the population classified themselves as of Asian, American Indian/Alaska Native, Native Hawaiian/Pacific Islander, or Other.

The study was based on the total of 659,313 individual 10-s ECG samples and the total of 7,350,733 individual beats accepted for the analysis. On average, there were 1,252 ± 220 and 1,269 ± 217 ECG segments, and 14,298 ± 2,983 and 13,825 ± 2,981 individual beats processed per each female and male subject, respectively (no statistical differences between sexes).

### 3.2 Comparisons of QRS-T angle expressions

#### 3.2.1 Differences between orthogonal lead systems

**FIGURE 1:**
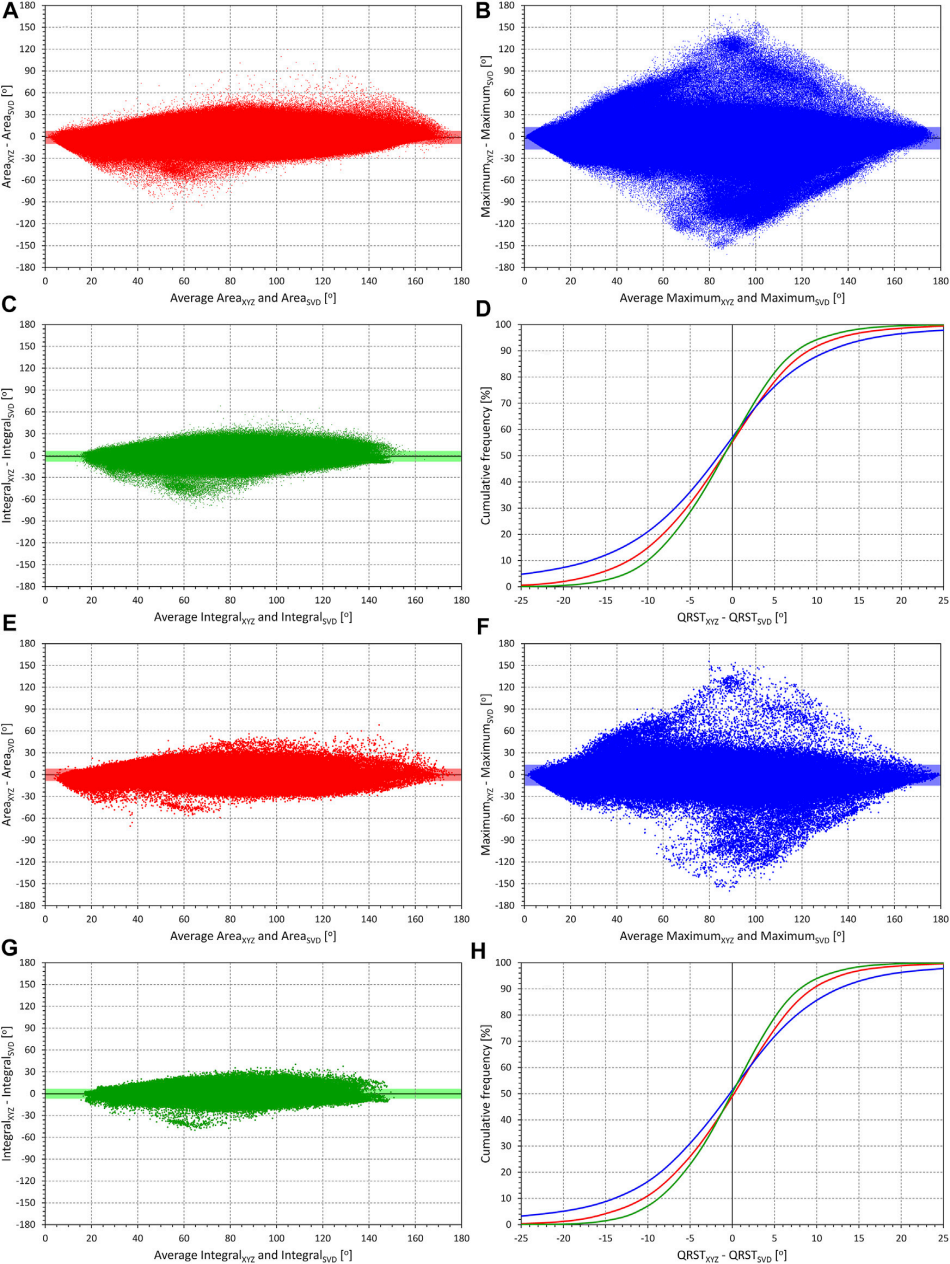
Comparison of QRS-T angle measurements in XYZ and SVD orthogonal projections. The matrix (XYZ) and the singular value decomposition (SVD) methods are used to derive orthogonal leads. In individual panels, the measurements in all subjects are pooled and the difference between XYZ and SVD angle expressions is plotted against their averaged value. The mean difference is shown by a bold horizontal line while the light-coloured band (along the horizontal axis) shows the spread of mean ± standard deviation. Panels **(A)** (comparisons of Area_XYZ_ and Area_SVD_), **(B)** (comparisons of Maximum_XYZ_ and Maximum_SVD_), and **(C)** (comparisons of Integral_XYZ_ and Integral_SVD_) show data derived from individual beats. Panel **(D)** shows cumulative distributions of the Method_XYZ_-Method_SVD_ values shown in panels **(A)**, **(B)**, and **(C)** (the colours of the graphs in this panel correspond to the colours of the scatter diagram panels). Panels **(E)**, **(F)**, and **(G)** show corresponding comparisons of the methods applied to representative waveforms of individual 10-s ECG segments. Panel **(H)** again shows the cumulative distributions of the method differences shown in panels **(E)**, **(F)**, and **(G)**. Note that the trapezoidal shape of the images (noticeable especially in panels **(B)** and **(F)** is caused by the measurements strictly between 0^o^ and 180^o^ (the difference of 180^o^ is only possible if one of the methods gives 0^o^ and the other 180^o^, in which case the average of the methods is 90^o^).

Scatter diagrams of the differences between QRS-T angle measurements made in conversion matrix based orthogonal XYZ leads and in orthogonal SVD-based leads are shown in Figure 1. Visual judgement of the images in Figure 1 suggests that the difference between the two orthogonal leads systems influenced the Integral method less than the other two methods while the Maximum method was influenced more than the other two methods. This applied to both the images based on individual ECG beats (top panels in Figure 1) and on representative median ECG waveforms (bottom panels in Figure 1).

**FIGURE 2:**
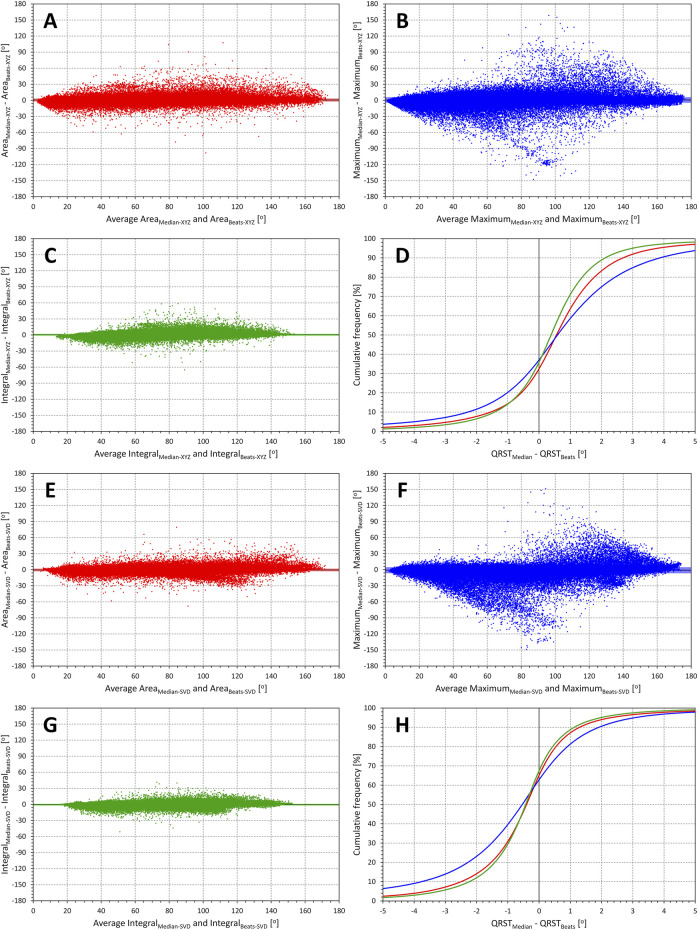
Comparisons of QRS-T angle measurements in individual beats and representative waveforms. Bland-Altman type of comparisons between QRS-T angle expressions measured at the representative waveform of 10-s ECG segments with the averages of the same angle expressions measured at individual beats of the same ECG segment. The layout of the figure and of the individual panels corresponds to that of Figure 1, with all the measurements in all study subjects pooled. In the method indicators, additional subscripts Median and Beats indicate measurement value derived from representative median waveform and obtained as an average of individual beats of the ECG segment, respectively. Panels (**A**,**B)** and **(C)** show the comparisons for the Area_XYZ_, Maximum_XYZ_, and Integral_XYZ_ angle measurements, respectively; panel **(D)** again shows the cumulative distributions of the measurement differences shown in panels **(A)**, **(B)**, and **(C)**. The same analysis of the results of methods Area_SVD_, Maximum_SVD_, and Integral_SVD_ is shown in panels **(E,F)** and **(G)**, respectively; corresponding cumulative distributions are shown in panel **(H)**.

**FIGURE 4:**
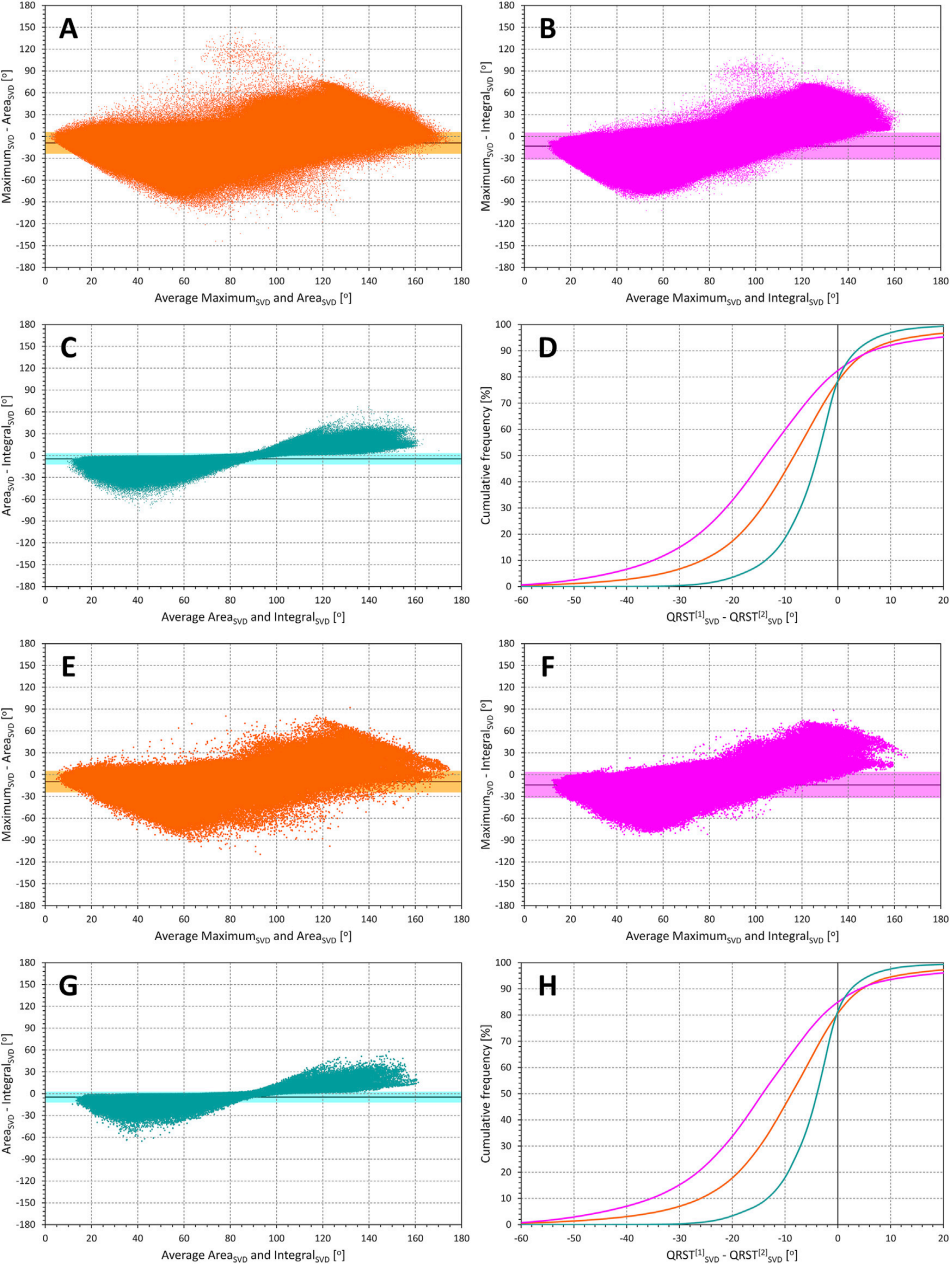
Comparisons of methods of QRS-T angle measurements in SVD orthogonal projections. Bland-Altman type of comparisons between different QRS-T angle methods applied to the orthogonal leads derived by singular value decomposition of the original 12-lead ECG signals. The layout of the Figure and the meaning of individual panels is the same as in Figure 3 but methods Maximum _SVD_, Area_SVD_, and Integral_SVD_ were analysed instead of Maximum_XYZ_, Area_XYZ_, and Integral_XYZ_, respectively.

**FIGURE 5:**
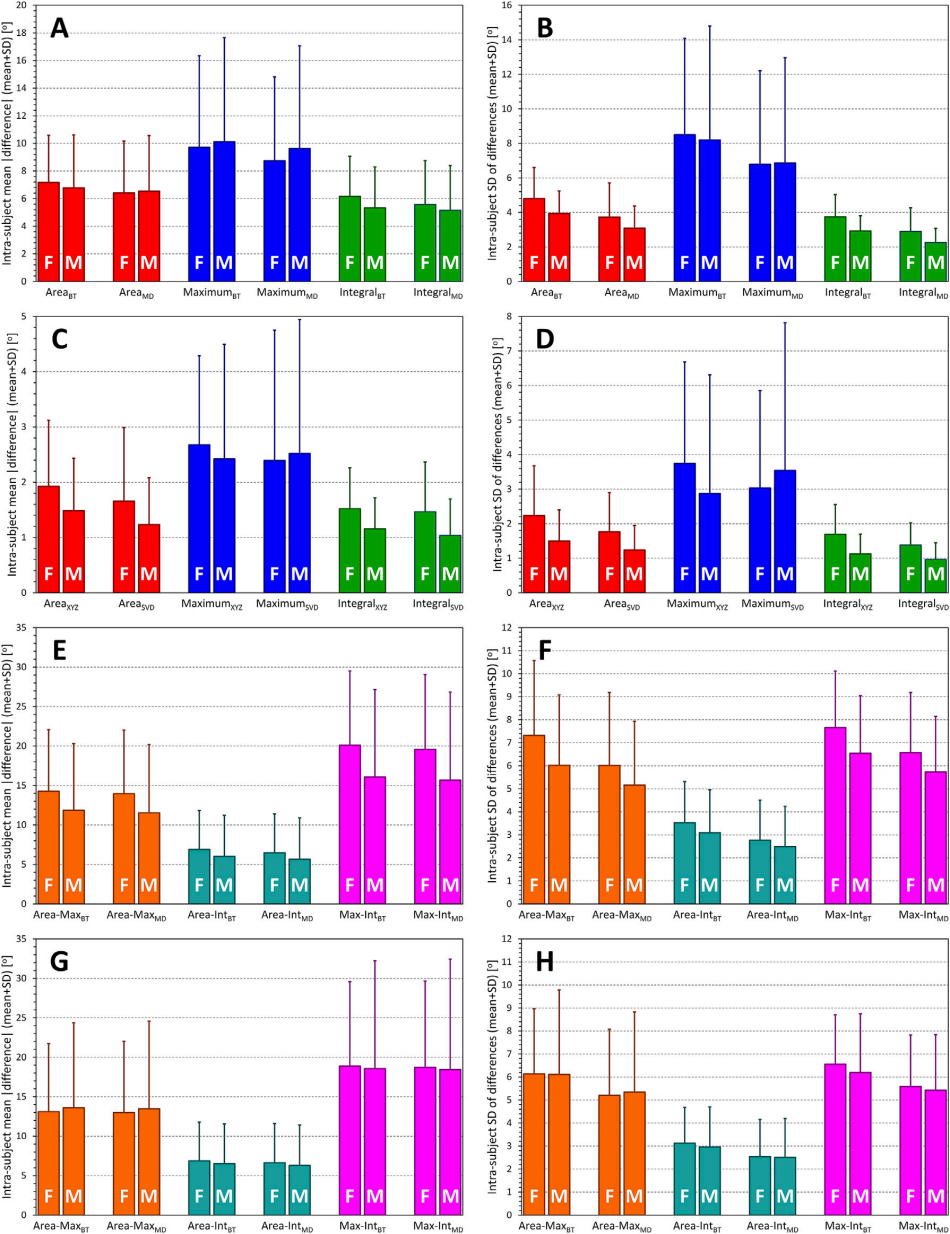
Statistical summaries of the differences between QRS-T angle measurements shown in Figures 1–4. Panel **(A)** shows the summary of intra-subject means of absolute values of the differences between the angle measurements in conversion matrix-derived XYZ orthogonal leads and SVD-derived optimised orthogonal leads; panel **(B)** shows the summary of intra-subject standard deviations of these differences. Panel **(C)** shows the summary of intra-subject means of absolute values of the differences between measurements in median waveforms of an ECG segment and the averages of measurements in individual beats of the same segment; panel **(D)** shows the summary of intra-subject standard deviations of these differences. Panels **(E)** and **(G)** show the summary of the intra-subject means of absolute values of the differences between different measurement methods applied to the matrix-derived XYZ orthogonal leads **(**panel **E)** and to the measurements SVD-derived optimised orthogonal leads **(**panel **G)**; panels **(F)** and **(H)** show the summary of intra-subject standard deviations of these differences. In each panel, statistics of female (F) and male (M) sub-populations are shown separately. See the text for the definition of measurement methods (Max – Maximum, Int – Integral). Subscripts Method_BT_ and Method_MD_ indicate measurements performed in individual beats and in the median waveforms, respectively. See the text for *p*-values of statistical comparisons.

**FIGURE 3:**
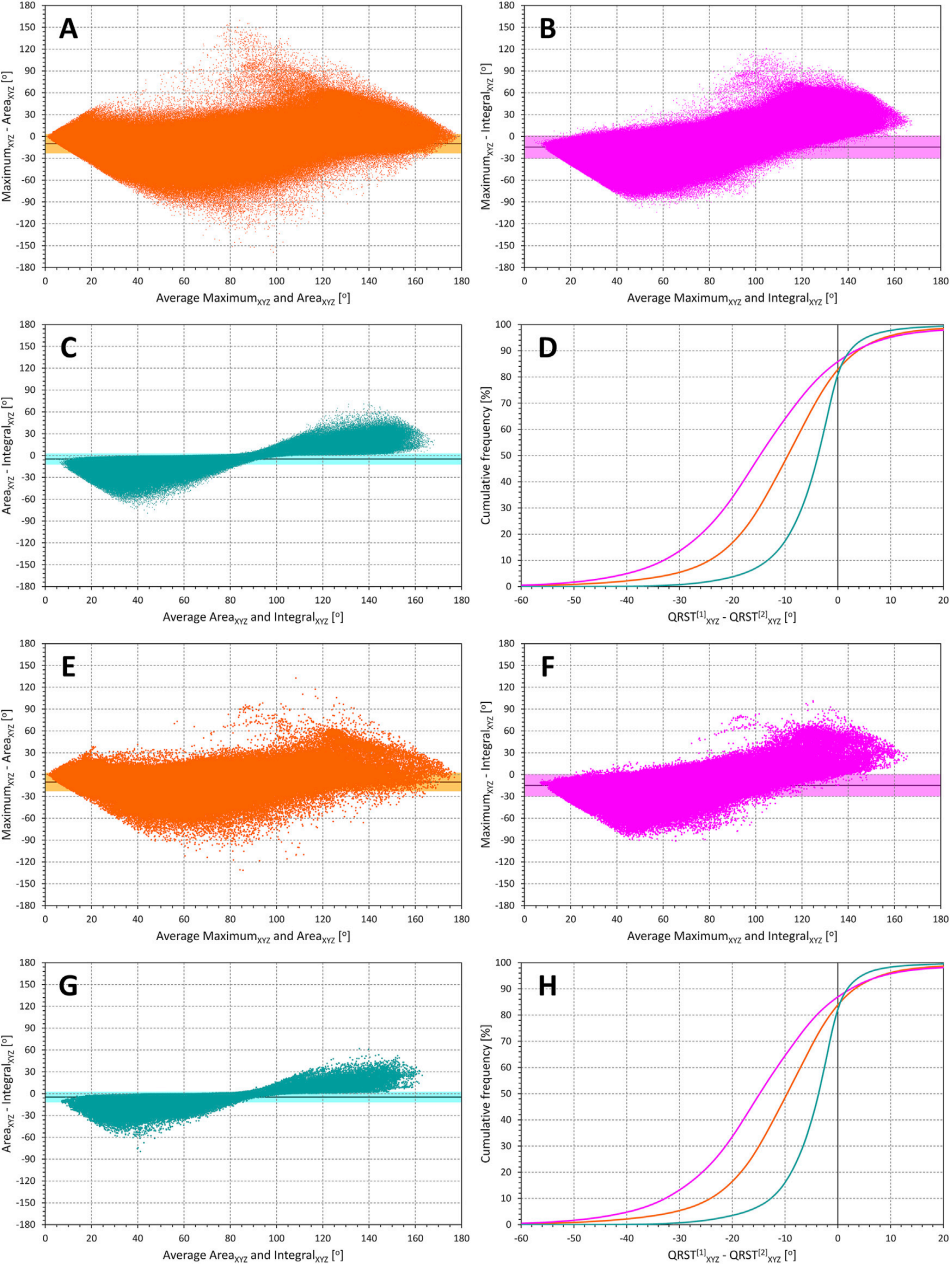
Comparisons of methods of QRS-T angle measurements in XYZ orthogonal projections. Bland-Altman type of comparisons between different QRS-T angle methods applied to the matrix-derived orthogonal leads XYZ. The layout of the figure and of the individual panels corresponds to that of Figure 1, with all the measurements in all study subjects pooled. Panels **(A–C)** shows the comparisons of Maximum_XYZ_ with Area_XYZ_, Maximum_XYZ_ with Integreal_XYZ_, and Area_XYZ_ with Integral_XYZ_, respectively, all values are derived from individual beat measurements. Panel **(D)** shows corresponding cumulative distributions, i.e., of pooled values Maximum_XYZ_─Area_XYZ_, Maximum_XYZ_─Integreal_XYZ_, and Area_XYZ_─Integral_XYZ_. Panels **(E–H)** show the same analysis applied to the measurements derived from representative median waveforms of 10-s ECG segments (again pooled over all study subjects).

This visual observation was confirmed statistically as shown in the top panels **A** and **B** of Figure 5. (Note that Figure 5 shows statistical summaries also of subsequent Figures 2–4.). All the differences between different QRS-T angle methods (and their applications to single beats and representative waveforms) seen in panel **A** of Figure 5 (intra-subject means of absolute values of the differences shown in Figure 1) were statistically significant (all *p* < 0.00001). The sex difference was only significant for the Integral method applied to individual beats (*p* = 0.0011). The intra-subject standard deviations of the differences shown in Figure 1 (see panel **B** of Figure 5) were also highly statistically different between different methods (and their applications to single beats and representative waveforms – *p* < 0.00001 for all). Sex differences seen in this panel of Figure 5 were also all statistically significantly different (*p* < 0.0001) except for both cases of the Maximum method.

#### 3.2.2 Differences between individual beats and representative waveforms

Similar visual comparisons between the methods are also seen in Figure 2 which shows the differences between QRS-T angle methods applied to representative waveforms and to the individual beats with results averaged over the same ECG segment. Regardless of whether the methods are applied to the matrix-based XYZ leads or to the SVD leads, the Integral method and the Maximum method appear least and most influenced, respectively.

Statistical analyses shown in panels **C** and **D** of Figure 5 confirm these observations. With the exception of the two variants (XYZ and SVD) of the Maximum method, all the between-method differences seen in panel **C** of Figure 5 were statistically significant (*p* < 0.0001 to *p* < 0.001). The results of the Maximum methods were also only those that showed no similar significant difference between females and males. The intra-subject standard deviations of the differences (panel **D** of Figure 5) showed the same results of comparisons with closely similar statistical significances.

#### 3.2.3 Differences between measurement algorithms

Scatter diagrams of the comparison between QRS-T angle calculation methods are shown in Figure 3 (XYZ matrix-based orthogonal leads) and 4 (SVD-based orthogonal leads). Generally, the Maximum method leads to lower values of the angle compared to the other two methods while on average, the Integral method leads to slightly higher results compared to the Area method. The statistical summaries of the absolute values and standard deviations of these differences are shown in panels **E** and **F** of Figure 5 (XYZ matrix-based orthogonal leads) and panels **G** and **H** of Figure 5 (SVD-based orthogonal leads). The difference between the Area and Integral methods was substantially and significantly lower compared to the Area—Maximum or Maximum—Integral differences and showed also lower intra-subject standard deviations (all *p* < 0.00001).

### 3.3 Heart rate dependency and hysteresis assessment

**FIGURE 6:**
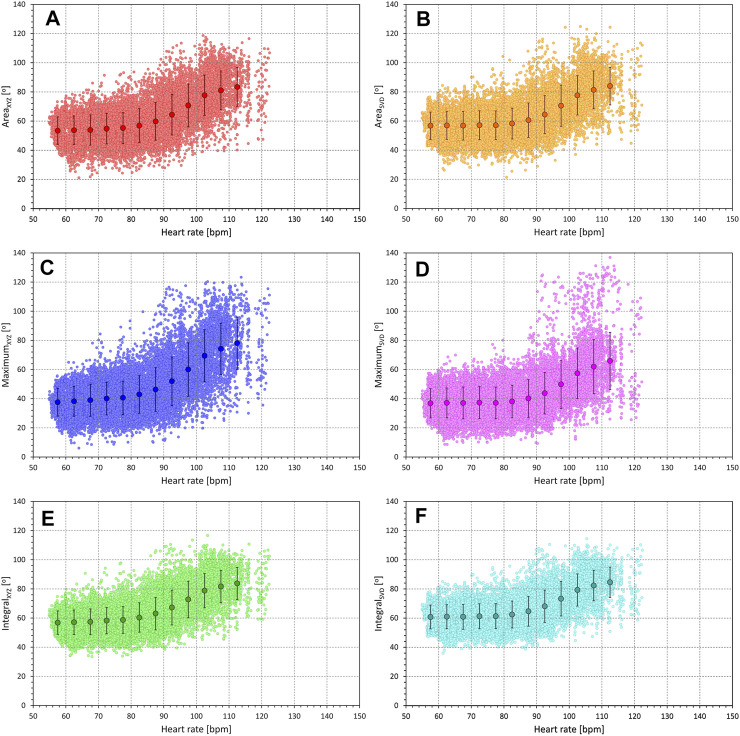
Example of the relationship of beat-based measurements of QRS-T angle and the heart rate measured over 1 min preceding each angle measurements. The data were obtained from the recordings of a 21.2-year-old female subject. Individual panels of the figure correspond to different QRS-T angle expressions; results corresponding to, Area_XYZ_, Area_SVD_, Maximum_XYZ_, Maximum_SVD_, Integral_XYZ_, and Integral_SVD_ angle expressions are shown in panels **(A–F)**, respectively. In each panel, the individual light-colour small marks correspond to the individual ECG beats data, the larger full-colour marks correspond to the averages of the QRS-T angle values in 5 beat per minute (BPM) bins, the error bars of the larger full-colour marks show the spread of ±1 standard deviation in the corresponding 5-BPM bins.

**FIGURE 7:**
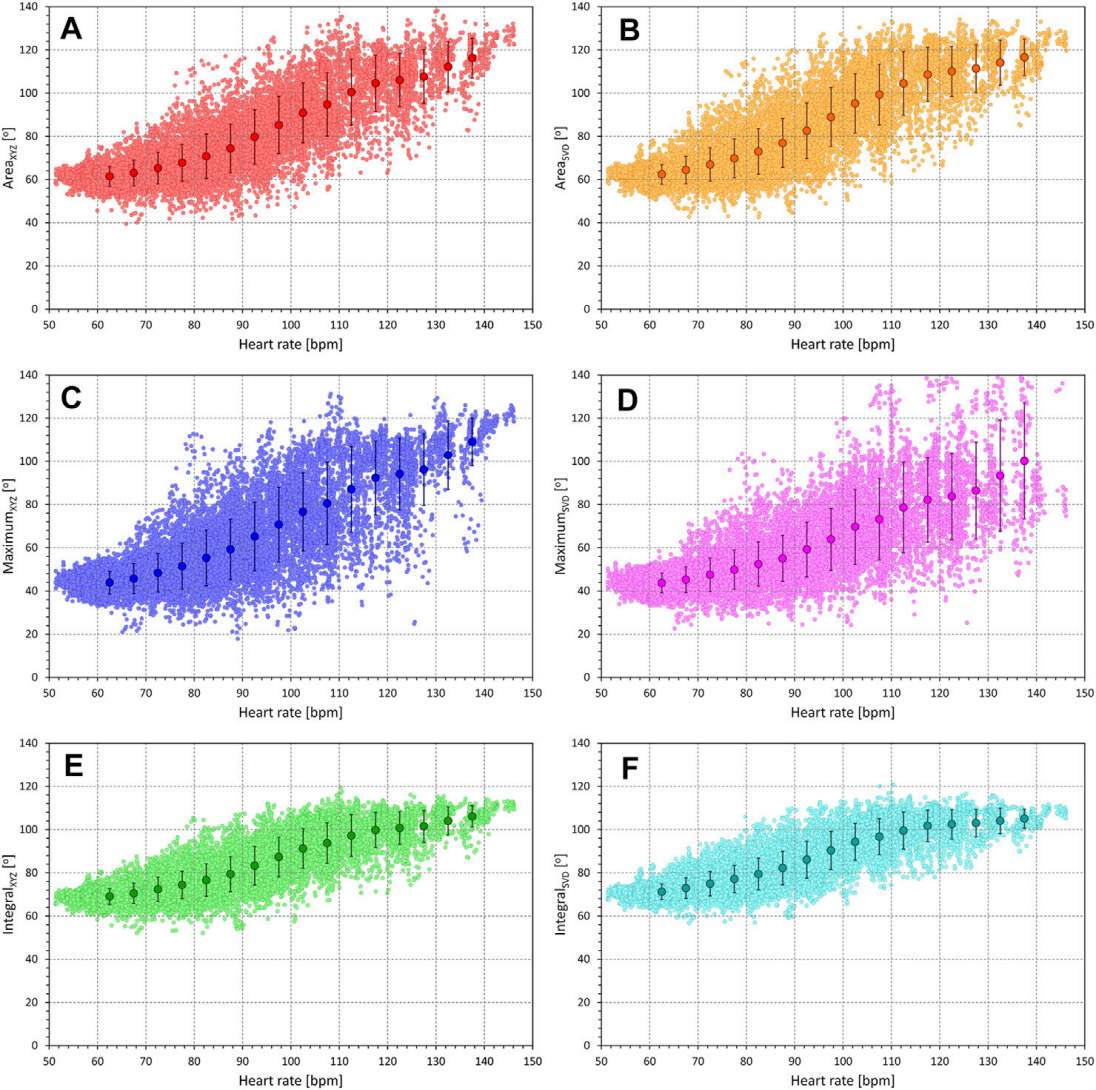
Example of the relationship of beat-based measurements of QRS-T angle and the heart rate measured over 1 min preceding each angle measurements. The data were obtained from the recordings of a 24.4-year-old male subject. The layout of the figure and the meaning of the symbols correspond to those in Figure 6.

Figures 6, 7 show examples of the relationship between QRS-T angle measurements and the underlying heart rate assessed in 1-min intervals preceding each single-beat angle measurements. The figures show clear correlation between the angles and the heart rate as well as an obvious non-linear nature of the relationship.

#### 3.3.1 Interval-based heart rate estimates

**FIGURE 8:**
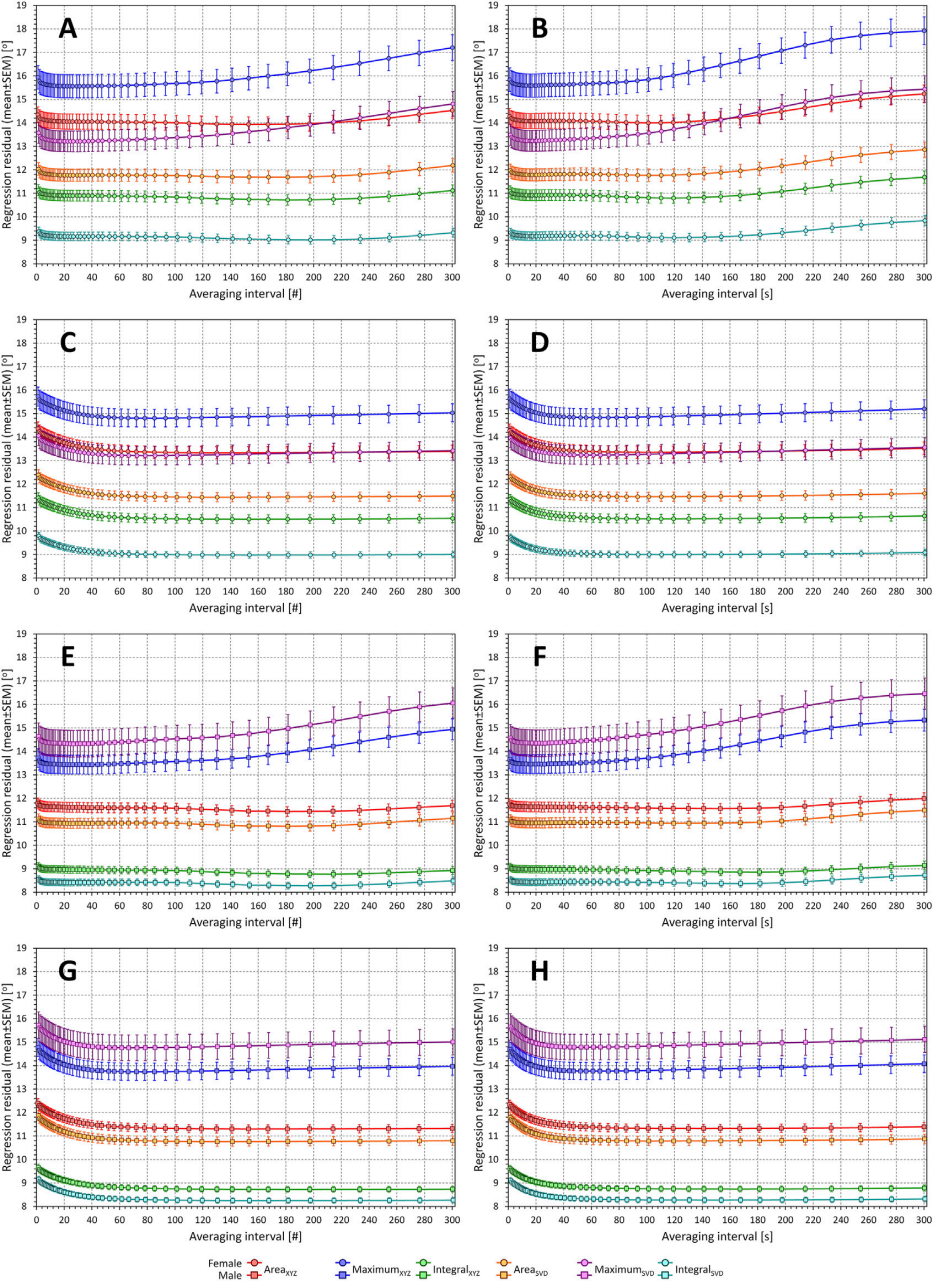
Regression residuals of QRS-T angle related to averaged preceding heart rate. In each panel, the individual graphs correspond to different QRS-T angle expressions and show the mean ± standard deviation of intra-subject residuals of second-degree polynomial regressions between QRS-T angle measurements and heart rates measured in preceding intervals of a given number of RR intervals [(#)—panels on the left] or a given number of seconds [(s)—panels on the right]. Panels **(A)** and **(B)** show the results in females with regressions involving only measurements preceded by variable heart rates; panels **(C)** and **(D)** show the results in females with regressions involving all measurements; panels **(E)** and **(F)** show the results in males with regressions involving only measurements preceded by variable heart rates; panels **(G)** and **(H)** show the results in males with regressions involving all measurements. Results related to the QRS-T angle expressions Area_XYZ_, Maximum_XYZ_, Integral_XYZ_, Area_SVD_, Maximum_SVD_, and Integral_SVD_ are shown in red, blue, green, amber, violet, and cyan, respectively. See the text for the definitions of the angle expressions.

The results of the experiments investigating interval-based QRS-T-angle/heart-rate hysteresis estimates are shown in Figure 8. Unexpectedly, the results do not suggest any physiologic range of heart-rate hysteresis estimates.

When tested considering only measurements preceded by variable heart rates, Maximum_XYZ_ and Maximum_SVD_ angle expressions reach closes fit to heart rate measured approximately over preceding 20 s or a similar number of RR intervals. With the other angle expressions, the result is much less clear since heart rate measurements of intervals longer than 20 s or 20 RR intervals do not appear to make any difference in the closeness of fit until approximately rate measurements over the preceding 3 min.

When testing the hysteresis effect in the complete data of each subject, the results are even less expected. When measuring the effect of heart rate assessed in an interval longer than approximately 30 s, the closeness of fit of the regressions between Area and Integral expressions and heart rate remains stable while the Maximum expressions show only minimal gradual increases of the regression residuals.

The intra-subject optimisation of the heart rate measurement was similarly inconclusive since no clearly defined minima of regression residuals were found in substantial majority of study subjects.

#### 3.3.2 Exponential decay estimates

**FIGURE 9:**
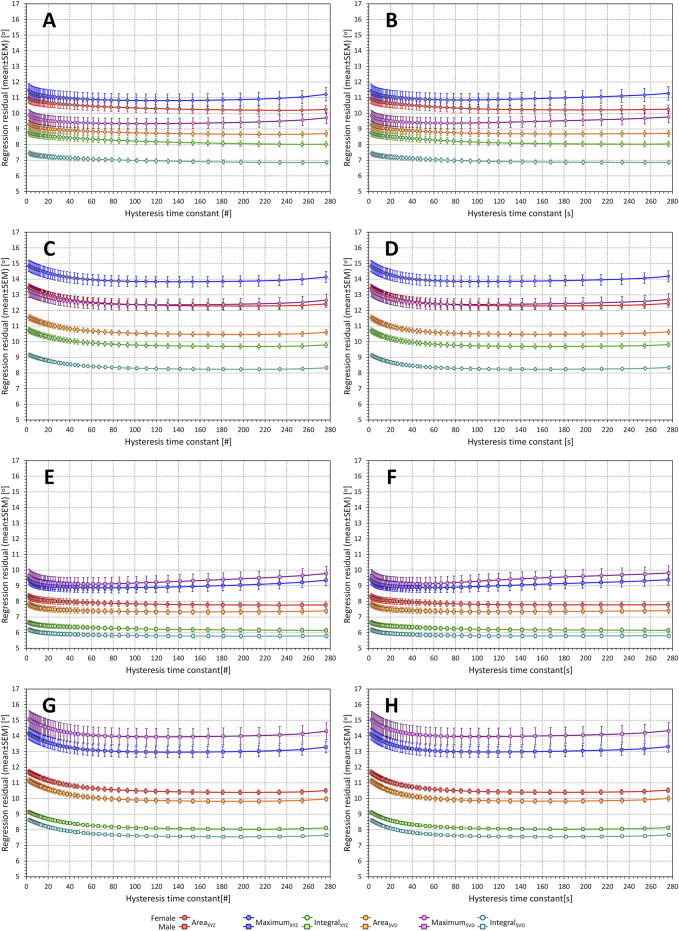
Regression residuals of QRS-T angle related to exponential decay of preceding heart rate. In each panel, the individual graphs correspond to different QRS-T angle expressions and show the mean ± standard deviation of intra-subject residuals of second-degree polynomial regressions between QRS-T angle measurements and heart rates derived by exponential decay hysteresis models with hysteresis constants of a given number of RR intervals [(#)—panels on the left] or a given number of seconds [(s)—panels on the right]. Panels **(A,B)** show the results in females with regressions involving only measurements preceded by variable heart rates; panels **(C,D)** show the results in females with regressions involving all measurements; panels **(E)** and **(F)** show the results in males with regressions involving only measurements preceded by variable heart rates; panels **(G)** and **(H)** show the results in males with regressions involving all measurements. Results related to the QRS-T angle expressions Area_XYZ_, Maximum_XYZ_, Integral_XYZ_, Area_SVD_, Maximum_SVD_, and Integral_SVD_ are shown in red, blue, green, amber, violet, and cyan, respectively. See the text for the definitions of the angle expressions.

These rather unexpected observations were replicated in experiment with exponential decay optimisation as seen in Figure 9. Only the Maximum expressions show some albeit very loosely defined minima in the search for optimum hysteresis constant. Both the Area and Integral expressions reach a stable level of regression residuals from approximately 30 to 40 s (panels in right part of Figure 9) or 30 to 40 beats (panels in the left part of Figure 9) onwards, regardless of whether the investigation is made using only beats preceded by variable heart rates or all study data.

Similar to the optimisation of heart rate measurements, no defined optimal hysteresis constants were found for almost all study subjects. With the Area and Integral expressions, the intra-subject golden cut searches converged to the maximum available scale of available data.

### 3.4 Physiologic characteristics

#### 3.4 1 Polynomial regression to heart rate

The optimum degree of polynomial regression between QRS-T angle and underlying heart rate was found to be ℘ = 2. Specifically, the proportions ${ℭ}^{\left(a,2\right)}/{ℭ}^{\left(a,1\right)}$ were below 0.95 in 17.7, 14.2, 13.2, 19.2, 14.4, and 14.0% of the population for Area_XYZ_, Maximum_XYZ_, Integral_XYZ_, Area_SVD_, Maximum_SVD_, and Integral_SVD_, respectively. The corresponding proportions of ${ℭ}^{\left(a,3\right)}/{ℭ}^{\left(a,2\right)}$ were below 0.95 in only 2.1, 2.9, 2.7, 2.5, 1.9, and 2.1% of the population.

Pooling female and male subjects together, the regression residuals of the second-degree polynomial regression between QRS-T angle and optimum (hysteresis-optimisation based) measurement of heart rate were 11.37 ± 3.26, 13.36 ± 5.55, 8.93 ± 2.38, 10.20 ± 2.71, 13.11 ± 7.28, and 7.97 ± 1.77° for Area_XYZ_, Maximum_XYZ_, Integral_XYZ_, Area_SVD_, Maximum_SVD_, and Integral_SVD_, respectively. (See also the subsequent section on intra-subject reproducibility for the comparison between sexes.)

#### 3.4.2 Sex differences

**FIGURE 10:**
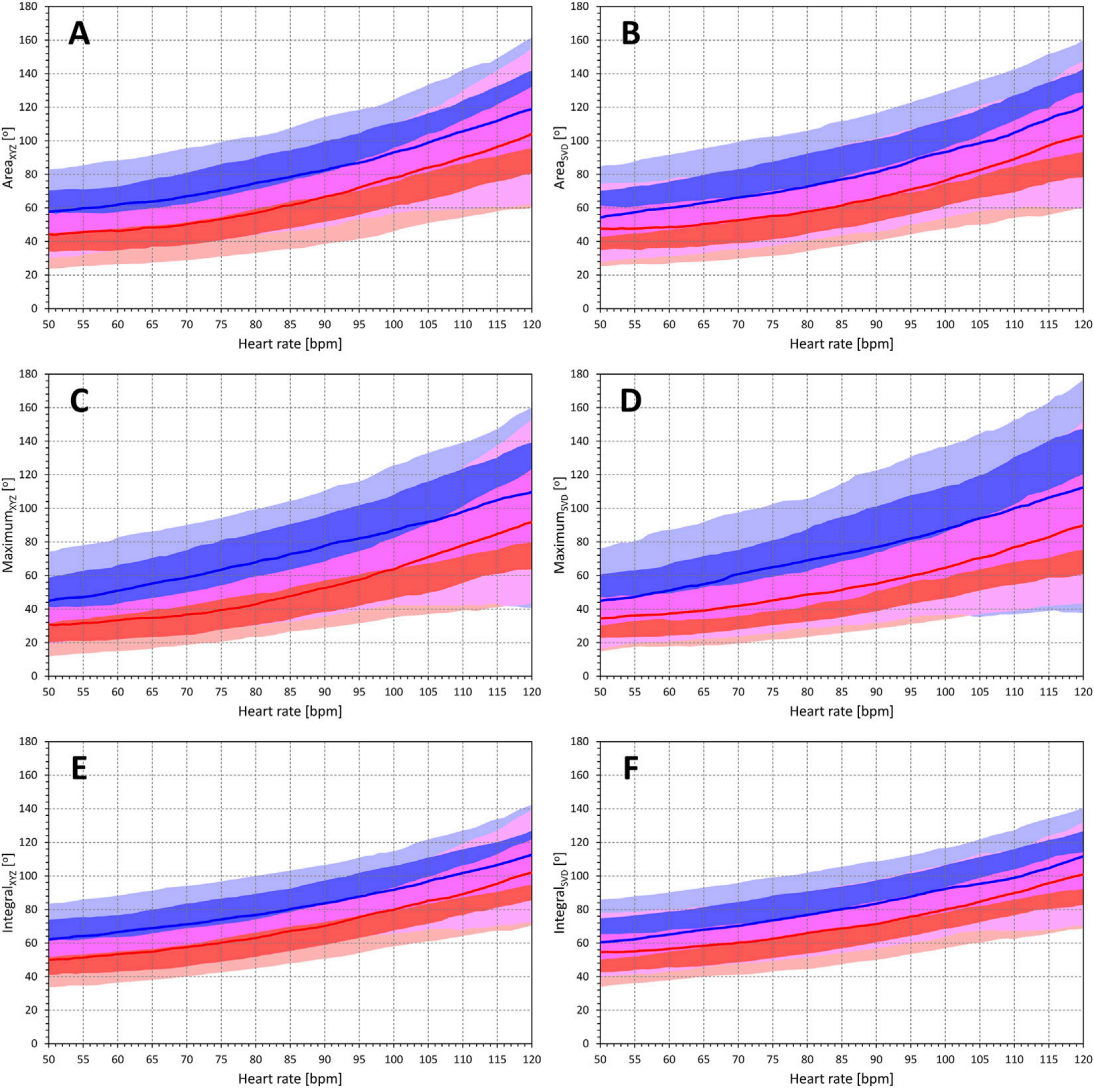
Population profiles of QRS-T angle relationship to underlying heart rate. Each panel of the figure corresponds to a different QRS-T angle expression and shows the summary of population distributions of intra-subject curvatures of second-degree polynomial regressions between QRS-T angle measurements and heart rate measured over the preceding 1 min. Bold red and blue lines show point-by-point median values of the regression curvatures in female and male subjects, respectively. The red and blue bands show the point-by-point inter-quartile ranges of the curvature values in females and males, respectively; the violet bands show the overlaps between the inter-quartile ranges between both sexes. The light red and light blue bands show the 10%–90% ranges of the curvature values in females and males, respectively; the light violet bands show the overlaps between the 10%–90% ranges between both sexes. The bands of inter-quartile ranges including their sex overlap are shown overlaying the 10%–90% bands. Panels **(A–F)** correspond to Area _XYZ_, Area_SVD_, Maximum_XYZ_, Maximum_SVD_, Integral_XYZ_, and Integral_SVD_ QRS-T angle expressions, respectively.

The intra-subject curvatures of second-degree polynomial regressions between QRS-T angle expressions and the underlying heart rate were used to construct images in Figure 10. Although, as seen in this Figure, there was a noticeable overlap between both sexes, the figure shows that irrespective of the underlying heart rate and irrespective of the QRS-T angle expression, females showed lower QRS-T angle than males. Dependent of the angle expression, values in females were approximately 10° to 20° lower than those in males.

**FIGURE 11:**
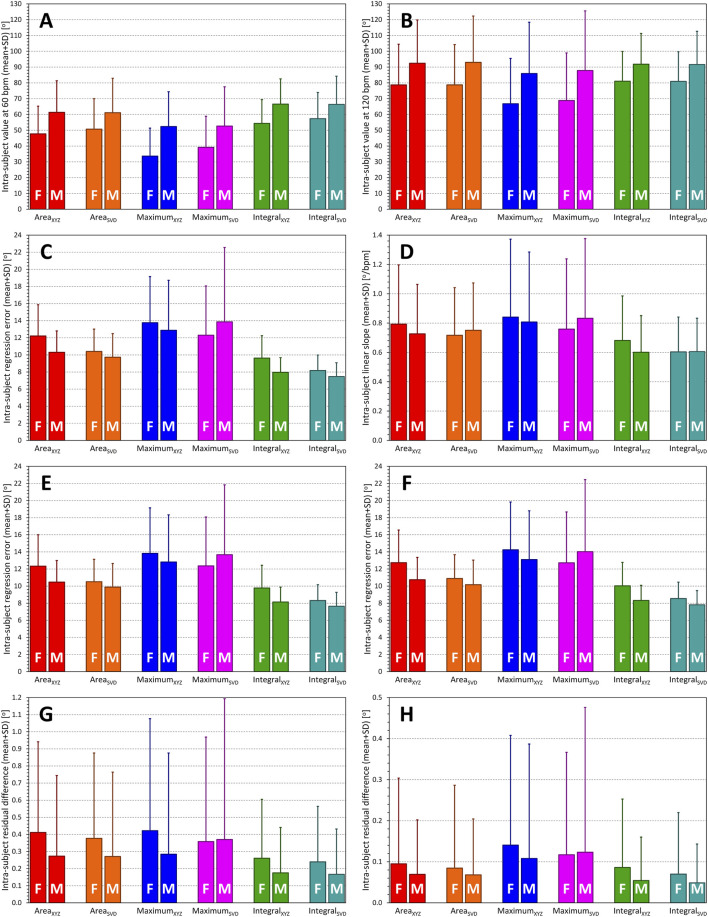
Summaries of QRS-T angle/rate regression residuals. Statistical evaluation of the characteristics of different QRS-T angle expressions (see the labels of the horizontal axes in each panel). Panels **(A,B)** show the intra-subject QRS-T angle values measured at the heart rate of 60 and 120 bpm, respectively (as derived by the second-degree polynomial regressions between QRS-T angle measurements and heart rate measured over the preceding 1 min). Panel **(C)** shows intra-subject residuals of the second-degree polynomial regression between QRS-T angle measurements and heart rate expressed by intra-subject optimum hysteresis model. Panel **(D)** shows intra-subject slopes of linear regressions between QRS-T angle measurements and heart rate measured during the preceding 1 min. Panels **(E,F)** show the intra-subject residuals of the second-degree (panel **E**) and linear (panel **F**) regressions between QRS-T angle measurements and heart rate measured over the preceding 1 min. Panels **(G,H)** show the decrease in regression residuals between second-degree and linear **(**panel **G)** and third-degree and second-degree **(**panel **H)** polynomial regression between QRS-T angle measurements and heart rate expressed by intra-subject optimum hysteresis model. In each panel, statistics of female (F) and male (M) sub-populations are shown separately. See the text for *p*-values of statistical comparisons.

Statistical evaluations of the sex differences are demonstrated in panels **A** and **B** of Figure 11 which show the sex-specific averages of QRS-T angles at heart rate of 60 and 120 bpm. All the sex differences shown in both these figure panels were highly statistically significant (*p* < 0.00001 for all sex comparisons).

Statistically significant differences were also noted between the different QRS-T angle expressions (corresponding to the measurement differences that were described previously). At the heart rate of 60 bpm (panel **A** of Figure 11), the results of the methods showed, on average, the following sequence: Maximum_XYZ_ < Maximum_SVD_ < Area_XYZ_ < Area_SVD_ < Integral_XYZ_ < Integral_SVD_ with all these differences between QRS-T angle expressions being highly statistically significant (*p* < 0.00001 in all pairs). At the heart rate of 120 bpm (panel **B** of Figure 11), the measurements by the Maximum_XYZ_ expression were, on average, still statistically significantly smaller than those by the Maximum_SVD_ expression (*p* = 0.007) which were, in turn, significantly smaller than the results by the other expressions (*p* < 0.00001). There were, however, no significant differences between the other angle expressions.

#### 3.4.3 Intra-subject reproducibility

Panel **C** of Figure 11 shows the regression residuals of second-degree polynomial regressions between QRS-T angles and the heart rate measurement derived by the intra-subject hysteresis optimisation. Despite the females showing lower QRS-T angle values, they also showed marginally but statistically significantly (*p*-value between 0.004 and <0.00001) higher regression residuals, i.e., lower intra-subject reproducibility of the QRS-T angle values “corrected for the underlying heart rate” when the Area or Integral expressions were used. The sex difference for the Maximum_XYZ_ expression did not reach statistical significance while the sex difference for the Maximum_SVD_ expression showed the opposite sex difference (*p* = 0.015).

More importantly, there were substantial disagreements between the different QRS-T angle expressions. In the total population, the highest residuals (13.31 ± 5.64°) were seen with Maximum_XYZ_ expression while the lowest residuals (7.82 ± 1.74°) were seen with the Integral_SVD_ expression. The averaged order of the residuals contrasted with the order measurement values since it was the opposite what was observed for the values measured at 60 bpm, that is the residuals were ordered Maximum_XYZ_ > Maximum_SVD_ > Area_XYZ_ > Area_SVD_ > Integral_XYZ_ > Integral_SVD_ with the individual step differences highly statistically significant (*p* < 0.00001) apart from the difference between Maximum_XYZ_ and Maximum_SVD_ which was only marginal (13.31 ± 5.64° vs. 13.11 ± 7.45°) and not statistically different.

#### 3.4.4 Curvatures of regression to heart rate

Panel **D** of Figure 11 shows the comparisons of intra-subject linear slopes between QRS-T angle expressions and the underlying heart rate. Sex differences are inconsistent but importantly, these slopes of the Integral expressions were significantly lower than those of the other expressions (*p* < 0.00001).

For comparison with the intra-subject reproducibility as illustrated in panel **C** of Figure 11, panels **E** and **F** of the same Figure show regression residuals of second-degree (panel **E**) and first degree, i.e., linear (panel **F**) regression analysis relating the QRS-T angle expressions to heart rate calculated based on a simple average of preceding 1-min RR intervals. While there are numerically slight (albeit statistically significant) increases in the displayed values from panel **C** to panel **E** as well as from panel **E** to panel **F**, the patterns are practically the same, highlighting the absence of any clearly detectable hysteresis-type delays between the changes of heart rate and of QRS-T angles.

Panel **G** of Figure 11 shows ${ℭ}^{\left(a,2\right)}-{ℭ}^{\left(a,1\right)}$ values, interpreted as the curvatures of the relationship between different QRS-T angle expressions and heart rate. As seen in the display, the QRS-T angle/heart-rate profiles were, on average, more curved in females compared to males (*p*-values between 0.01 and 0.001) for all angle expressions except of Maximum_SVD_.

Finally, panel **H** of Figure 11 shows ${ℭ}^{\left(a,3\right)}-{ℭ}^{\left(a,2\right)}$ values. Similar trends as observed in panel **G** can be seen, although without statistical significances. The panel also shows that these residual differences were very tiny compared to those shown in panel **G** (note the difference in the vertical axes of both panels).

None of the characteristics summarised in Figure 11 appeared to be correlated with age.

#### 3.4.5 Relationship to race, body mass index and lean body mass

The race categories others that African or White Caucasian origin were too infrequent for any meaningful analysis. Therefore, the race comparison was only possible between subjects of African and White Caucasian origin. Neither statistically significant differences nor trends towards borderline statistical differences were found.

**FIGURE 12:**
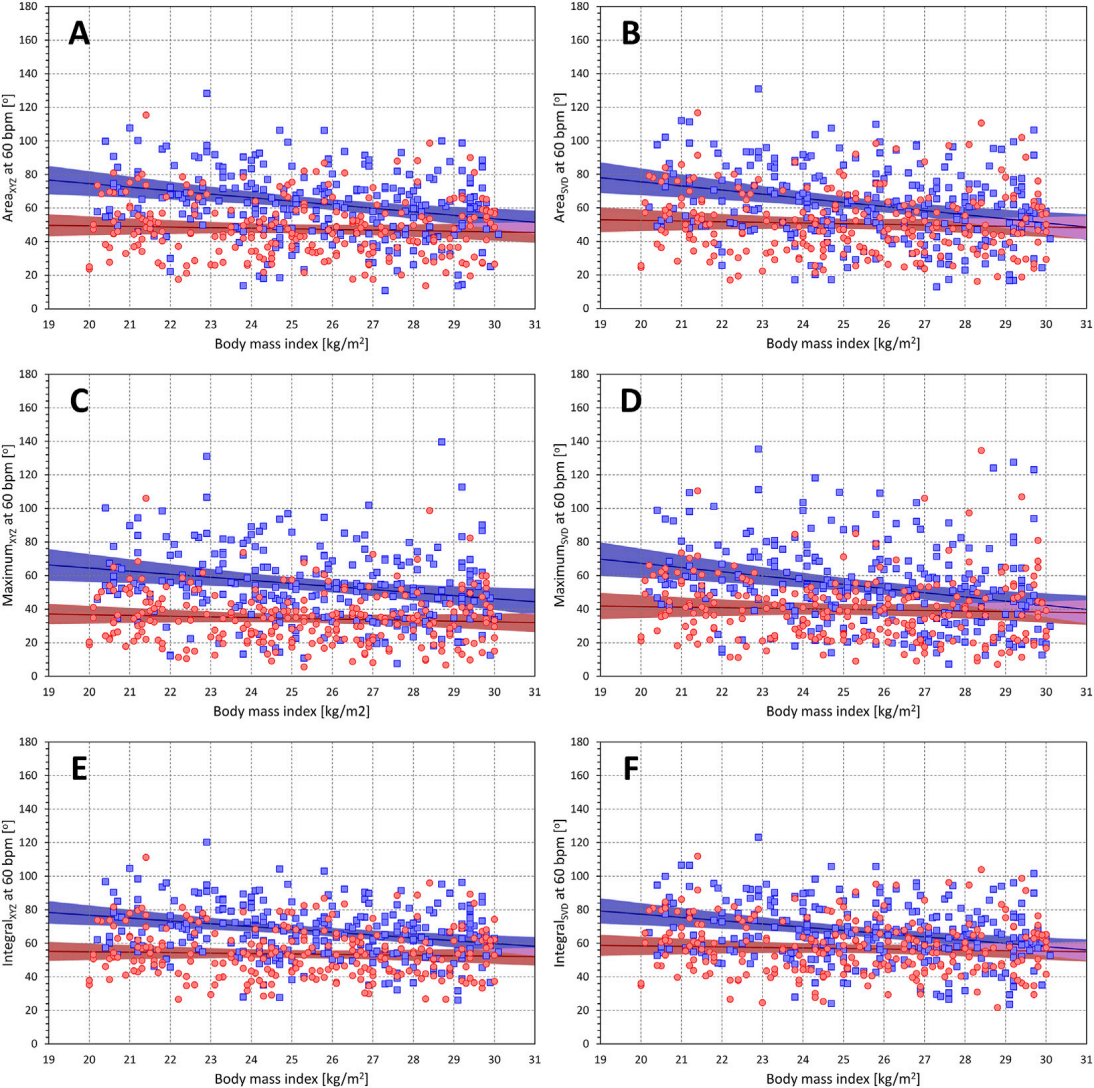
Relationship of QRS-T angle (projections to heart rate of 60 bpm) to body mass index. The different panels of the figure show scatter diagrams between body mass index and subject-specific projections of different QRS-T angle expressions to the heart rate of 60 bpm. Panels **(A–F)** show QRS-T angle data of Area_XYZ_, Area_SVD_, Maximum_XYZ_, Maximum_SVD_, Integral_XYZ_, and Integral_SVD_, respectively. In each panel, the red circles and blue squares show the data of female and male subjects, respectively. The red and blue bold lines are linear regression of QRS-T angle to body mass index in female and male sub-populations, respectively. The light red and light blue areas are the 95% confidence bands of the sex-specific regressions, the light violet areas are the overlaps between the regression confidence bands of both sexes.

**FIGURE 13:**
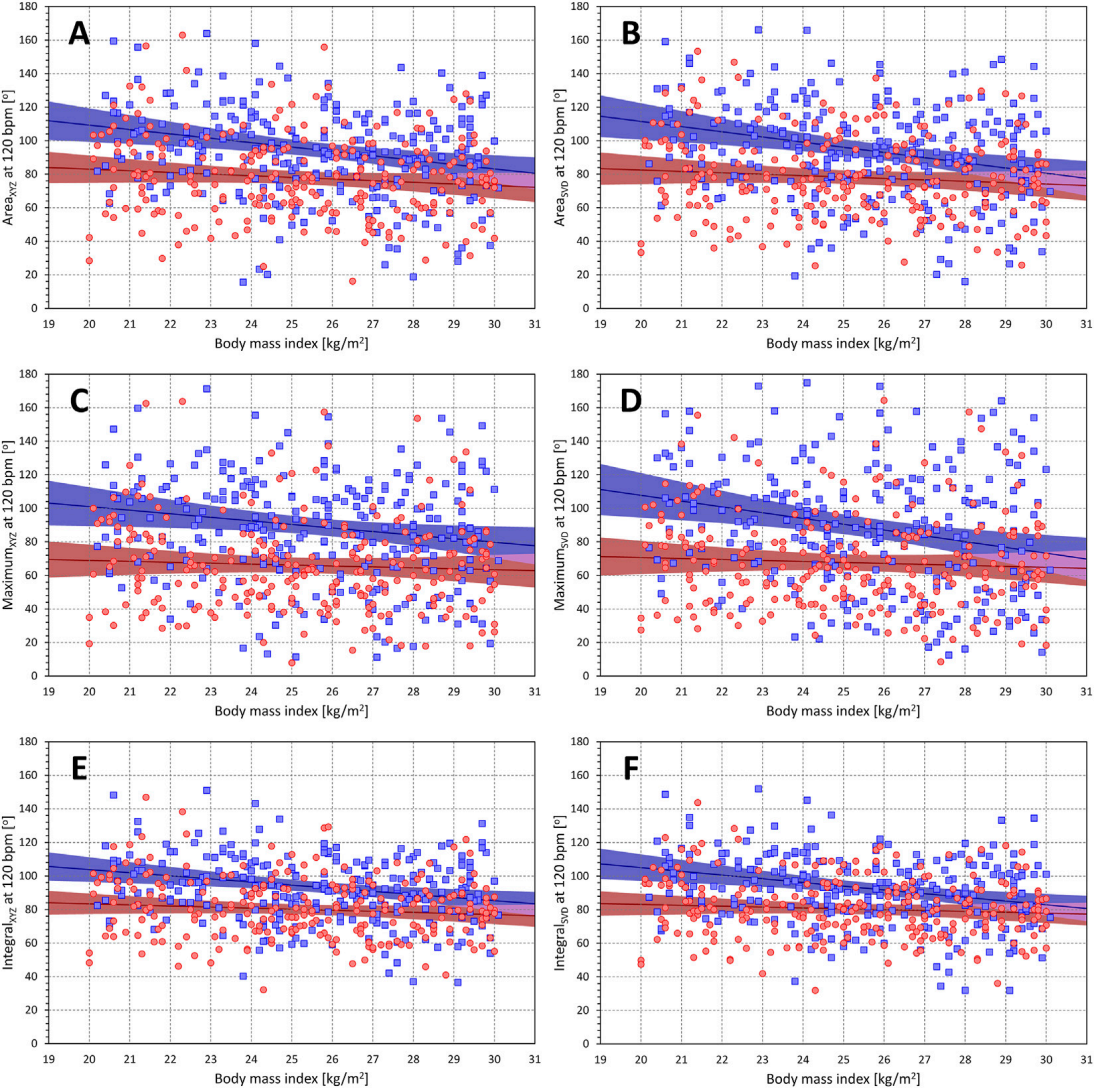
Relationship of QRS-T angle (projections to heart rate of 120 bpm) to body mass index. The different panels of the figure show scatter diagrams between body mass index and subject-specific projections of different QRS-T angle expressions to the heart rate of 120 bpm. The layout of the figure and the meaning of the individual symbols is the same as in Figure 12.

The projections of the ${\text{Θ}}^{\left(a\right)}$ values at heart rate of 60 and 120 bmp were borderline correlated with BMI in females (*p*-values of the significance of the Pearson correlations ranged between 0.039 and 0.337) and were systematically significantly correlated with BMI in males (*p*-values of the significance of the Pearson correlations ranged between 0.012 and <0.0001). The corresponding scatter diagrams and linear regressions of the projected ${\text{Θ}}^{\left(a\right)}$ values are shown in Figure 12 (projections to the heart rate of 60 bpm) and Figure 13 (projections to the heart rate of 120 bpm). In females, the correlation coefficients relating the BMI to the two projections of different QRS-T angle expressions ranged between −0.136 and −0.062, and the projected ${\text{Θ}}^{\left(a\right)}$ values were decreasing by between 0.32 and 1.13 degrees per one unit of BMI. In males, the correlations ranged between -0.260 and −0.155 and the projected ${\text{Θ}}^{\left(a\right)}$ values were decreasing by between 1.27 and 2.48 degrees per one unit of BMI.

No such significant or borderline significant correlations were found when studying the relationship to LBM (investigations in females and males made separately).

## 4 Discussion

The study led to several observations of which three appeared of physiologic and practical importance.

First, despite dealing with very substantial datasets, we were unable to determine any clear hysteresis-type delay between heart rate changes and QRS-T angle changes. The most likely explanation of this observation is that QRS-T angle does not react to heart rate (e.g., in a similar way as the duration of the QT interval does) but that it is influenced directly by mechanisms and regulatory processes that simultaneously influence the heart rate. Second, we show important practical differences between the methods of the QRS-T angle measurement. Third, we confirm that under normal circumstances, female hearts show lesser differences between QRS and T wave loop orientations.

### 4.1 Cardiac autonomic and neurohumoral status

Our first observation likely suggests that the QRS-T angle is directly influenced by cardiac autonomic and neurohumoral status (Malik and Camm, 1990; Task Force, 1996; Puglisi et al., 2013; MacDonald et al., 2020) rather than driven by the frequency of ventricular depolarisations. The initial 30–40 s period of regression instability might be interpreted as a predominant sympathetic influence with less clear vagal modulations.

If this observation is confirmed in independent data, it would offer a substantial advance in the assessment of cardiac autonomic status at the level of ventricular myocardium, e.g., through the paraventricular ganglia (Zaglia and Mongillo, 2017), rather than at the level of sinus node. Measurement of cardiac autonomic responsiveness at the sinus nodal level has long been available by the heart rate variability (HRV) techniques (Task Force, 1996; Malik et al., 2019) and although new approaches are still being developed, most with the aim of more accurately distinguishing sympathetic and parasympathetic influence (Valenza et al., 2018), all the heart-period-based techniques fail when sinus nodal periodicity is absent (e.g., in atrial fibrillation) or disturbed (e.g. by pacing, frequent ectopic activity, sinoatrial blocks, etc.). In addition to these situations, it would also be beneficial to accompany HRV-based assessment of cardiac autonomic status by independent technique, especially if the results could be obtained on beat-to-beat basis which is the case with QRS-T angle. This would offer advances to cardiac risk stratification as well as to early assessment systemic neuropathies (May et al., 2018). Linking the QRS-T angle to cardiac sympathetic status would also help explaining its risk prediction properties.

The possibility that the assessment of QRS-T angle might serve this purpose is supported by some of the previous observations. The predictive value of QRS-T angle was reported additive to HRV-based risk stratification (Poulikakos et al., 2018) in a multivariate analysis of the follow-up cardiac events and mortality in a population of end-stage renal disease patients on haemodialysis. QRS-T angle was also reported predictive of mortality in atrial fibrillation patients who had an automatic cardioverter-defibrillator implanted for primary prophylactic reasons (Hnatkova et al., 2022). At the same time, however, our observations of the absence of any clearly measurable time lag between heart rate changes and QRS-T angle changes, while in agreement with previous observations of rapid changes (Hnatkova et al., 2010), are somewhat at odds with the report by Kenttä et al. (2010) who described hysteresis-type differences between RR interval changes and QRS-T angle changes during exercise testing in a relatively small study of healthy volunteers undergoing exercise testing. Their report of hysteresis-type patterns is rather descriptive than quantitative and therefore not necessary in complete disagreement with our findings. They also used measurement techniques close to the Maximum method which, as described subsequently, we consider to be the least suitable of the measurement possibilities and which is also influenced by postural and other changes of T wave morphology. The conjecture that the QRS-T angle might reflect cardiac sympathetic status might also be potentially challenged by the lack of the relationship to age that we were unable to document. However, this needs to be considered together with the appreciable spread of intra-subject values as seen in Figures 6, 7, and with the limited range of ages of investigated subjects.

Further studies are needed to verify our conjecture of the direct sympathetic influence on QRS-T angle. Among others, atrial pacing during electrophysiology studies with abrupt changes of the stimulation rate [i.e., investigations similar to the seminal studies of QT/RR hysteresis (Franz et al., 1988; Lau et al., 1988)] would be helpful to elucidate whether the observed relationship of the QRS-T angle to heart rate is driven solely by the changes in the ventricular depolarisation frequency or whether inducement of autonomic changes is needed to influence the angle. Spectral analysis of beat-to-beat measurement of the QRS-T angle during distinctly different autonomic conditions (Pomeranz et al., 1985; Hnatkova et al., 2019) might also help in the assessment of our conjecture, especially if accompanied by the estimates of the coherence between the QRS-T angle and RR interval spectra.

### 4.2 Technology of QRS-T angle measurement

Our second observation relates to the differences between the methods used for QRS-T angle assessment. Not only were the results by the three methods significantly different but the reproducibility of the methods was also substantially and significantly distinct. Of the three algorithmic methods tested, the Maximum approach appeared least reliable. Regardless of which orthogonal lead system was used, the results of the Maximum method differed from the other two methods both in the terms of mean values but also in terms of the spread, i.e., standard deviations of the differences. The intra-beat and intra-median waveform differences between Maximum_XYZ_ and Maximum_SVD_ results were also significantly larger compared to the other two methods and, in terms of the relationship to the underlying heart rate, the Maximum method also appeared to show significantly lower intra-subject reproducibility which is well known to be of substantial practical importance for any risk assessment method (Malik et al., 1992). This poor performance of the Maximum method agrees with lesser risk prediction power reported in independent clinical data (Hnatkova et al., 2018).

The Area method is well known (van Oosterom, 2014) but, as far as we are aware, the Integral method has not been reported previously. At the same time, the philosophy of these two methods is similar. It is easy to see that in the Area method, the QRS complex and T wave loop 3-dimensional orientations are derived as averages of vectors moving around the QRS complex and T wave loops, with the contribution of each vector weighted by its magnitude. The Integral method takes this philosophy one step forward and computes the QRS-T angle and an average of all angles between all pairs of vectors moving around the QRS complex and T wave loops, with the contribution of each vector pairs weighted by the product of their magnitudes. It is therefore not surprising that these two methods were significantly closer to each other compared to their differences from the Maximum method. The significantly tighter intra-subject reproducibility of the Integral method offers clear advantage over the Area method.

With both Area and Integral computation methods, we have also observed reduction of intra-subject regression residuals (i.e., increase in intra-subject reproducibility) when using SVD-based rather than conversion matrix-based XYZ orthogonal leads. This is not surprising since SVD leads to optimally constructed orthogonal leads for individual QRS-T patterns of each beat or each representative waveform. On the contrary, using the same conversion matrix for all ECG is likely to lead to some signal loss of the orthogonal components that might be of importance for the valid assessment of the angle.

Considering all this together, the study suggests that the Integral_SVD_ is the optimum expression of the 3-dimensional QRS-T angle. It showed not only the tightest intra-subject reproducibility but also the closest correspondence between the averaged measurements of individual beats within a 10-s ECG segment and the measurement performed at the representative waveform of the same segment.

The six different QRS-T angle expressions that we have investigated are naturally not the only possibilities. In addition to simplistic measurements that can be performed “by hand” using standard 12-lead ECG images (Rautaharju et al., 2007), different conversion matrices might be used (Schreurs et al., 2010), and even simple quasi-orthogonal leads considered (Cortez et al., 2014). Combination of different approaches is also possible. The very first studies that showed the clinical usefulness of the QRS-T angle (Acar et al., 1999; Zabel et al., 2000) utilised the so-called TCRT measurement of the angle which was, in principle, a combination of the Integral (from the QRS side) and of the Maximum (from the T wave side) measurement algorithms.

### 4.3 Orthogonal lead systems

There is a principal difference between the orthogonal lead systems that we used for the conversion of the 12-lead ECGs into 3-dimensional representations in which the QRS-T spatial angles can be computed. The conversion to the XYZ leads was based on a published transformation matrix (Guldenring et al., 2012) that was originally derived from simultaneous recordings of standard 12-lead Mason-Likar recordings and Frank orthogonal electrocardiograms. In this system, the orientation of the XYZ axes is determined anatomically and the conversion thus approximates signals that would have been collected, for a given ECG, in the standard right → left, front → back, and head → foot directions. In this sense, the XYZ leads are anatomically orthogonal and, dependent on the position of the heart in the thorax, are related to the physical geometry of the organ. On the contrary, the S1, S2, and S3 leads derived from the SVD transformation do not have defined relationship to the physical geometry of the thorax or the organ but are made algebraically orthogonal by the SVD-based matrix manipulation. SVD also produces a conversion matrix to approximate the original ECG leads from the S1, S2, and S3 signals (Acar and Köymen, 1999; Hnatkova et al., 2021) but these conversion matrices differ for different ECGs.

Because of the transformation differences, the orthogonal system of leads S1, S2, and S3 cannot be considered as a simple rotation of the XYZ axes. Both the SVD and the XYZ conversion matrix utilise the information from all original ECG leads (both transformations involve only the 8 algebraically independent leads I, II, V1, V2, …, V6). However, since both transformations act differently, the 3-dimensional morphology of the QRS complex and T wave loops are not identical (even if spatially rotated) and thus, the angles measured between these loops are not the same. Nevertheless, as shown in Figure 1, the differences between the orthogonal representations are not large when considering the Area and the Integral methods (the Maximum method is clearly affected by the differences in the morphology of the QRS complex and T wave loops in both representations). This is because the information in the signals of the different ECG leads differs only little from the projection of “ideal” 3-dimensional QRS and T wave loops. Indeed, it was shown (Acar et al., 1999; Hnatkova et al., 2021) that in normal ECGs, the power of the ECG signal beyond three orthogonal leads is in small single percentages. Hence, although the 3-dimensional morphology of the QRS complex and T wave loops is not the same in both representations, it is not diametrically different either.

As already discussed, the results of the study give some preference to the SVD-based conversion to the orthogonal leads. This likely because both the XYZ matrix conversion and the SVD-based orthogonal representation involve certain level of numerical imprecision. The XYZ matric conversion applies the same transformation matrix to every ECG. Although the matrix was derived from a regression analysis involving more than 500 separate ECG signals (Guldenring et al., 2012), it cannot be assumed that the relationship between the 12-lead signals and true orthogonal Frank leads would be the same in every subject and every recording. The SVD-based conversion to S1, S2, and S3 leads omits the signals that project into the 4th to 8th algebraic orthogonal dimensions. The tight intra-subject reproducibility and beat-to-beat stability of the Integral_SVD_ method might suggest that, on average, the errors of the 3-dimensional XYZ reconstruction might be marginally larger than the errors due to the omission of the higher algebraic components derived by SVD.

### 4.4 Sex differences

While differences between sexes have been described before (Smetana et al., 2004), the projected differences between females and males (as seen in Figure 10) appeared, somewhat surprisingly, practically constant at different heart rates. The sex differences were also confirmed with all QRS-T angle expressions, although the extent of the differences appeared larger with the Maximum method and smaller with the Integral method. Both the sex and the influence by the different expressions were likely contributed by the sex differences in ECG morphology (Macfarlane, 2020). Despite the lower mean values of the QRS-T values in females, we also observed marginally larger spread of the values in the female sub-population and lower intra-subject reproducibility in females.

From a practical point of view, using the same normal value and/or the same dichotomy of the QRS-T angle for both females and males seems inappropriate for clinical risk assessment studies. Such studies either need to use different normality limits or, preferably, include sex of the patients as well as heart rates of the analysed QRS-T angle values in multivariable analyses. Our data also suggest that the Integral_SVD_ expression might be the optimum way of assessing the QRS-T angle in both females and males. With this expression, sex differences of around 10° might be expected.

Similar to other electrocardiographic sex differences (Linde et al., 2018), it seems reasonable to hypothesise that the QRS-T angle is influenced by sex hormones. Further studies of the phenomenon, e.g., of the differences during menstrual cycle or of pubertal development (Andršová et al., 2019), might provide more detailed understanding. Wider QRS-T angles in males might also be contributed by wider spread of repolarisation across ventricular myocardium that is seen in longer Tpeak-Tend intervals in males (Andršová et al., 2020).

### 4.5 Relation to BMI

Although statistically significant in males and borderline significant in females, the decreases in the QRS-T angles with increasing BMI were only modest. Perhaps, this is partially because per protocol of the source clinical studies, only subjects with BMI between 20 and 30 were included. Still, two possible explanations for such a population trend might be proposed.

First, even a marginal increase in BMI might signify modest thickening in the chest fatty layers that likely act as electrical capacitors with slight effects on the difference between epicardial potentials and surface ECG signals. Investigations of ECGs recorded in substantially obese subjects might be helpful to investigate this phenomenon further. Second, even within this narrow range of the BMI values, their differences might be related to the different levels of athletic training and physical activity that was not systematically assessed in the study subjects. Such differences might manifest by different levels of autonomic activity (Guo et al., 1999) as well as by moderate increases in ventricular mass (Augustine and Howard, 2018). Both factors might affect the values of the QRS-T angles.

When using the QRS-T angle assessment in future risk-prediction studies, relation to BMI might need to be considered, especially when dealing with very lean or markedly obese patients.

### 4.6 Limitations

Limitations of our study also need to be considered. Importantly, as our data were obtained in healthy volunteers participating at clinical pharmacology studies, we are not able to relate our QRS-T angle measurement to any cardiac risk and/or follow-up events. While the Maximum and Area method have previously been shown to be potent risk predictors, the Integral method still needs to be investigated in this way although the so-called TCRT method of the very first risk-studies of the angle is a combination of the integral approach applied to the QRS complex with the maximum approach applied to the T wave (Zabel et al., 2000; Zabel and Malik, 2001; Malik et al., 2004; Hnatkova et al., 2018). The restriction to healthy subjects also does not allow us to comment on whether the same or similar findings would be found in patient populations recorded under different clinical conditions (e.g., heart failure patients, survivors of acute myocardial infarction, heart transplant recipients, etc.). Nevertheless, assessment methods that are lesser reproducible in healthy volunteers are unlikely more stable in patients with ECG abnormalities. The SVD analysis of the source 12-lead ECGs would also allow to measure the QRS-T angle in more than three orthogonal directions. All three analytical methods might be applied in SVD-derived orthogonal systems of four or more dimensions although it is questionable whether the gradually decreasing amplitudes of the ECG components in the additional dimensions would change the 3-dimensional measurements noticeably. The age ranges of the study population were some 35 years wide and did not include any subjects over 60 years of age. Nevertheless, an obvious and statistically significant age relationship of heart rate variability, QT/RR hysteresis profiles, and of individually corrected QTc intervals was previously observed over similar or even narrower age ranges (Andršová et al., 2022; Toman et al., 2022). Finally, the data of the study were derived from ECG recordings obtained using Mason-Likar electrode positions. As far as we are aware, no direct comparison is available of QRS-T angles measured in simultaneously recorded ECGs using standard and Mason-Likar electrode positions. Nevertheless, when the same dichotomy limits were applied to the TCRT data derived from recordings of standard and Mason-Likar electrode configurations, similarly strong risk prediction was obtained (Hnatkova et al., 2022). We therefore consider it likely that the method comparisons presented here would also apply to standard ECG recordings. The demographic measurements of body weight and height as well as the ages of the subjects were determined objectively. The study did not include any sex-transversal subjects and the sex differentiation was therefore also objective. On the contrary, the race classification was self-declarative and no genetic or other data are available to confirm the subjective race declarations objectively.

## 5 Conclusion

Despite these limitations, the study shows that it is plausible to speculate that spatial QRS-T angle measurement might allow direct assessment of cardiac autonomic responsiveness at the ventricular level. Further evaluations and confirmations of this hypothesis are needed but if confirmed, it would not only explain the risk-prediction properties of the angle but also allow employing the angle measurement in focused profiling of cardiovascular risk. The study also shows that the newly proposed Integral measurement of the angle offers increased measurement stability especially if the measurement is performed using the SVD-derived orthogonal leads optimised for each analysed ECG recording. Finally, the study confirms sex differences in physiologic QRS-T angle measurement with values in females lower than values in males irrespective of the heart rate at which the measurement is performed.

## Data Availability

The raw data supporting the conclusions of this article will be made available by the authors, without undue reservation, but pending the approval by the sponsors of the source clinical studies.
